# Reduced changes in protein compared to mRNA levels across non-proliferating tissues

**DOI:** 10.1186/s12864-017-3683-9

**Published:** 2017-04-18

**Authors:** Kobi Perl, Kathy Ushakov, Yair Pozniak, Ofer Yizhar-Barnea, Yoni Bhonker, Shaked Shivatzki, Tamar Geiger, Karen B. Avraham, Ron Shamir

**Affiliations:** 1 Department of Human Molecular Genetics and Biochemistry, Sackler Faculty of Medicine and Sagol School of Neuroscience, Tel Aviv University, Tel Aviv, 6997801 Israel; 2 Blavatnik School of Computer Science, Tel Aviv University, Tel Aviv, 6997801 Israel

**Keywords:** Inner ear, Cochlea, Mass spectrometry, RNA-seq, Translation

## Abstract

**Background:**

The quantitative relations between RNA and protein are fundamental to biology and are still not fully understood. Across taxa, it was demonstrated that the protein-to-mRNA ratio in steady state varies in a direction that lessens the change in protein levels as a result of changes in the transcript abundance. Evidence for this behavior in tissues is sparse. We tested this phenomenon in new data that we produced for the mouse auditory system, and in previously published tissue datasets. A joint analysis of the transcriptome and proteome was performed across four datasets: inner-ear mouse tissues, mouse organ tissues, lymphoblastoid primate samples and human cancer cell lines.

**Results:**

We show that the protein levels are more conserved than the mRNA levels in all datasets, and that changes in transcription are associated with translational changes that exert opposite effects on the final protein level, in all tissues except cancer. Finally, we observe that some functions are enriched in the inner ear on the mRNA level but not in protein.

**Conclusions:**

We suggest that partial buffering between transcription and translation ensures that proteins can be made rapidly in response to a stimulus. Accounting for the buffering can improve the prediction of protein levels from mRNA levels.

**Electronic supplementary material:**

The online version of this article (doi:10.1186/s12864-017-3683-9) contains supplementary material, which is available to authorized users.

## Background

The correlation between expression levels of protein and mRNA in mammals is relatively low, with a Pearson correlation coefficient of ~0.40 [1, 2]. Suggested explanations for this low correlation include post-transcriptional regulation and measurement noise [1]. This low correlation makes it difficult to integrate protein and mRNA data. Tools for this integration are sparse and not yet adopted by the bioinformatics community (reviewed in [3]). Initial findings from such tools suggest that the transcriptional and the translational regulation evolved independently, except in the rare occasions where strong selection in favor of correlation was present [4]. However, such claims are based on data from perturbed systems, where the observed discordance between the transcriptome and the proteome is strongly affected by the lack of temporal synchronization between the transcriptional and translational regulation levels [5]. In this study we focus on the connection of mRNA and protein levels in non-proliferating tissues, through the example of the mammalian inner ear. By performing joint analysis of RNA-seq and protein mass spectrometry (MS) data from the mouse cochlea and vestibule, we aimed to shed light on the regulation of these two expression levels, identify genes that are mainly regulated in one system, and infer their general features. The two tissues are quite similar in structure, but have distinct roles in hearing and balance. This allows us to ask questions about the contribution of each of these two systems of regulation with respect to different cellular roles.

We will refer to a gene’s protein level divided by its transcript level as the gene’s protein-transcript ratio or PTR, also called the gene’s translation efficiency [6]. We note that this measure is affected by both translation and protein degradation rates, and under steady-state conditions it should be equal to the ratio of the rates [7]. It was observed that across taxa, protein levels are more conserved than mRNA levels [8], although some exceptions exist [9]. Also, it was noticed that differences in protein levels between primates are less common than differences in mRNA levels [10]. While PTR was claimed to be highly conserved between tissues for each given protein [11], it was demonstrated that it somewhat varies between tissues in a direction that buffers or compensates for the change in protein levels from changes in the transcript abundance [7], similar to what was shown across taxa. However, these observations originated from a small number of tissues, and were based mainly on regression coefficients that are affected by regression dilution bias [12]. In the first part of this study we will ask whether this phenomenon is evident in our mammalian inner ear data, and in previously obtained transcriptomic and proteomic data from different tissues. We will then use our discoveries to improve the prediction of protein levels from mRNA levels.

Many experiments only measure transcript abundance in a tissue and use it as a proxy for protein levels. Previous articles that predicted protein levels from mRNA [6, 13] did not use PTR measured in other tissues, and relied mainly on sequence related features; they reached a correlation of 0.75 between the predicted and the observed levels. It has been suggested to use the average PTRs measured in other tissues in order to predict the protein levels for the tissue in question [8]. This assumes the PTR of a gene is constant across tissues. We suggest, instead, a model that assigns a higher PTR in a tissue where the mRNA level is lower.

In the second part of this study we use functional analysis to compare differential expression across tissues in mRNA and protein. We give examples where inner-ear tissues maintain different levels of mRNA and similar levels of protein at rest, and hypothesize that this is done in preparation for a stimulus.

## Results

Previous examinations of mRNA-protein relationships were mainly performed in yeast and in cancer cell lines. Aiming to examine these associations in non-transformed cells and differentiated tissue samples, we analyzed four different paired datasets of mRNA and protein. For the first dataset we generated transcriptomic and proteomics data from the cochlea and vestibule of mouse inner ear (dataset termed EAR). The three other datasets were publicly available: (i) multiple mouse tissues (termed MMT; RNA-seq [14] and proteomics [15]); (ii) primate lymphoblastoid cells (PRIMATE; [10]); and (iii) a panel of human cancer cell lines (NCI60; transcription microarrays [16] and proteomics [17]). The results obtained for the NCI60 dataset were compared with those obtained for datasets of non-transformed cells.

The EAR RNA-seq analysis identified 39,178 genes, 14,722 of which have at least one read per million in three or more of the samples and were included in the analysis. MS analysis identified 7244 proteins (Additional file 1: Table S1). Six thousand eight hundred thirty-two genes were common between the two tissues.

The MMT dataset contains mRNA and protein levels taken from mouse tissues. In the proteomic data [15], the stable isotope labeling with amino acids in cell culture (SILAC) technique was used as an internal standard for relative quantification of proteins across 28 mouse tissues. We used five tissues that had both mRNA and protein data: brain, cerebellum, heart, kidney, and liver. There were three proteomic samples for brain (cortex, medulla, and midbrain) and two for kidney (cortex and medulla), and we weighted the samples’ contribution by the volumes of the subregions to obtain the tissue protein levels. mRNA measurements had three replicates per tissue, and six for the brain.

The PRIMATE dataset includes transcriptomics (RNA-seq) and proteomics (SILAC-based) data from lymphoblastoid cell lines (LCLs) derived from five human, five chimpanzee, and five rhesus macaque. The species is analogous in the subsequent analysis to the tissue. We downloaded the data from [10], and processed it as described in the article, to obtain expression levels of (orthologous) genes that have at least three measurements from each of the three species, for both mRNA (12,079 genes) and protein (3688 genes). Three thousand three hundred ninety-four genes were common between mRNA and protein.

NCI60 is a panel of 59 diverse human cancer cell lines. The type of cancer is analogous in the subsequent analysis to the tissue. We note that we do not necessarily expect to see the same phenomena in cancer cell lines as in healthy tissues, due to the pathological state of the tissues, and as the cell lines of the same cancer are different samples and not real replicates as the healthy tissues. One manifestation of these differences is a lesser ability to separate NCI60 samples based on their origin, compared to the EAR and MMT datasets. Indeed, multi-dimensional scaling (MDS) plots show better separation of the latter datasets on both mRNA and protein levels, even between very similar tissues (Additional file 2: Figure S1). Moreover, poor results were reported when hierarchical clustering was used to perform such a separation for breast, ovary, renal, and prostate cancers using proteomic data [17].

We refer to the tissue type (in EAR and MMT), species (in PRIMATE), or cancer type (in NCI60) as a *group*. We refer to samples of the same group as *replicates*. We refer to mRNA and protein as *domains*.

### Protein levels are more conserved than mRNA levels

mRNA and protein levels were log_2_ -transformed, and averaged across all samples from the same group, disregarding missing values. A comparison of the proteomic and transcriptomic data showed, in agreement with previous studies [18], that the overall dynamic range of mRNA is significantly lower than protein, as marked by a higher variability in protein expression compared with mRNA in all datasets (Additional file 2: Figure S2, Additional file 3: Table S2).

We calculated protein-mRNA correlations for each group (see Additional file 2: Supplementary Methods). The average correlations between the two layers were 0.58, 0.44, 0.42, and 0.42 for the EAR, MMT, NCI60 and PRIMATE datasets, respectively, similar to the mRNA-protein correlations reported in the literature [1]. Then, we calculated correlations between pairs of groups for mRNA and protein separately. We observed that in all datasets, all the protein-protein and the mRNA-mRNA correlations between groups were higher than the protein-mRNA correlations within each group (Additional file 2: Figure S3). This last trend was somewhat weaker in the MMT dataset, which includes less similar tissues.

**Fig. 1:**
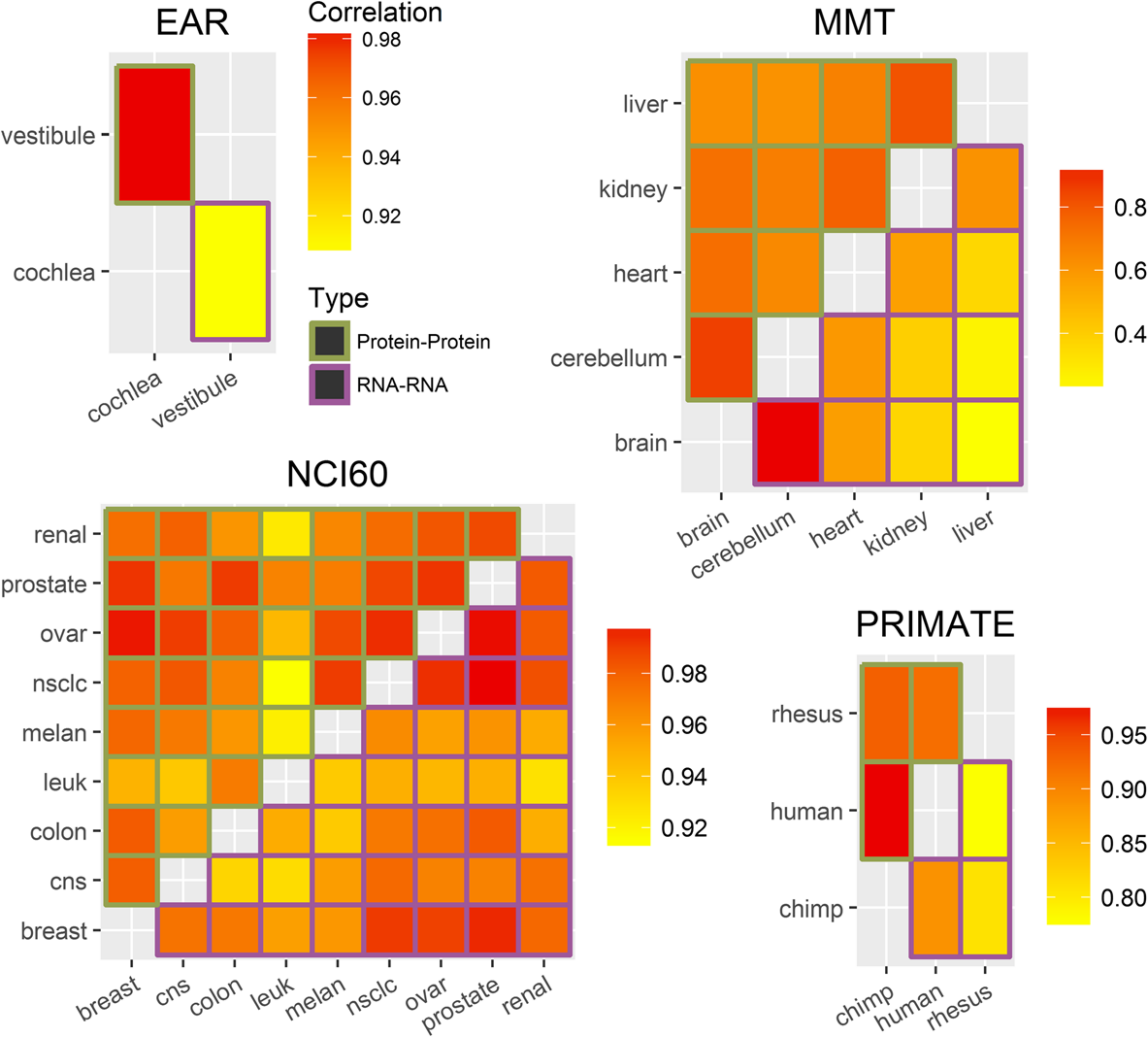
Protein and mRNA correlation between group pairs. Each subfigure describes the correlation between expression levels of different groups in one dataset. The *upper and lower triangles* show the protein-protein and mRNA-mRNA correlations between groups, respectively. *Darker color* corresponds to higher correlation. Pearson’s correlation coefficients (r) were corrected using Spearman’s method except in the MMT dataset (due to the lack of replicates in protein). See Additional file 2: Figure S3 for intra group protein-mRNA correlations

Figure 1 demonstrates a comparison of the correlation between group pairs in each dataset. For the EAR dataset the correlation in the protein between the cochlea and the vestibule is higher than the correlation in the mRNA (0.97 versus 0.94). This is also the case for the PRIMATE dataset (3/3 pairs), the MMT dataset (9/10 pairs), and the NCI60 dataset (24/36). For the MMT and NCI60 datasets the protein correlations were significantly higher (*p − value*s = 2.9 × 10^−3^ and 8.0 × 10^−3^ respectively, Wilcoxon signed-rank test). To account for some of the platform differences between RNA-seq and mass spectrometry, which manifest in higher correlation between replicates of RNA-seq (Additional file 2: Supplementary Results, Figure S4), we applied the Spearman’s correction in our calculations, except for MMT where it was inapplicable (see correction example in Additional file 2: Figure S5; explanation for MMT in Supplementary Results).

### PTRs vary in a direction that reduces protein divergence

The higher correlation between pairs of groups in the protein domain suggests that changes in transcription between tissues are coupled to protein-level changes that exert opposite effects on the final protein level, hence producing higher similarity between groups. We call the phenomenon of reduced (“compressed”) change in protein levels compared to the change in mRNA levels *buffering*. Spangenberg et al. showed this phenomenon in the initial phases of adipocyte differentiation of adipose-derived human mesenchymal stem cells, by comparing differentiating cells at two time points [7]. Regressing the fold change (FC) of the protein levels to the FC of the mRNA levels on a log-log scale led to the observation of a slope lower than 1, or, in other words, range compression between protein FC and mRNA FC. They hypothesized that a trend of lower PTR with increasing mRNA levels is the cause.

**Fig. 2:**
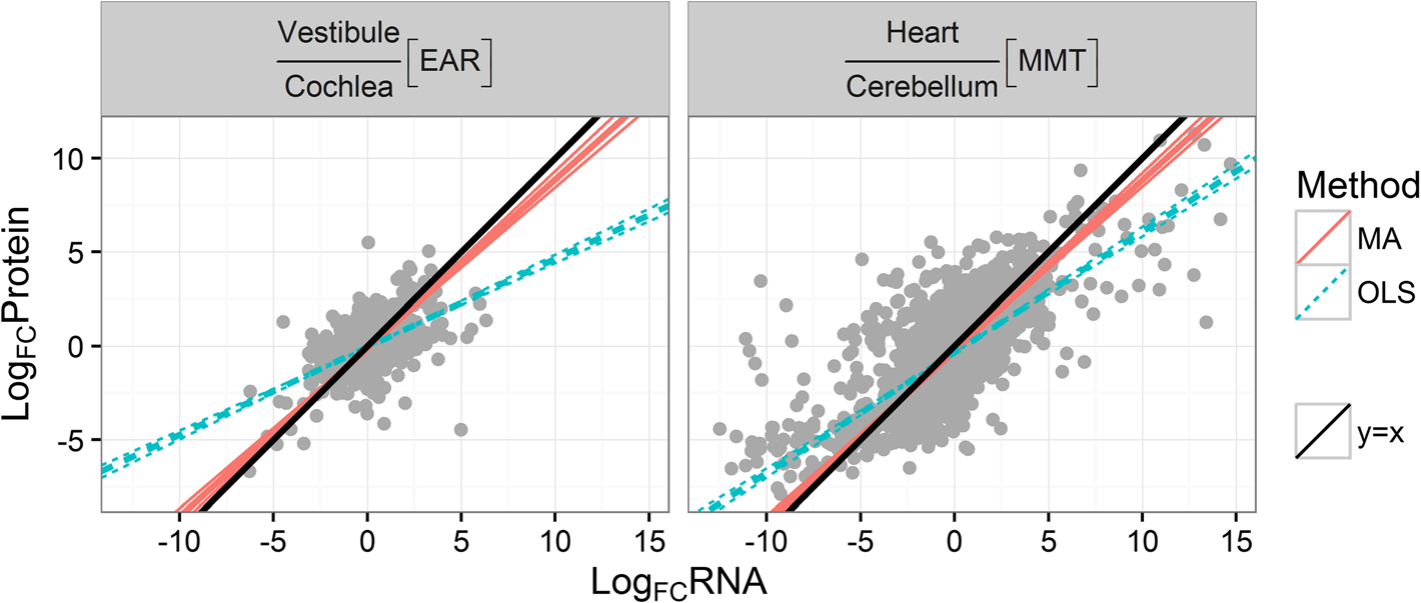
Examples of range compression. Comparing either the cochlea and the vestibule EAR tissues (*left*), or the heart and the cerebellum MMT tissues (*right*), the protein fold changes (*y-axis*) were regressed on the mRNA fold changes (*x-axis*). The fitted regression lines using ordinary least squares (OLS, *red*, *solid*) and major axis regression (MA, *blue, dashed*) were plotted, along with their 95% confidence interval (*thinner lines*). The black line is y = x. Both OLS and MA slopes are significantly lower than 1, suggesting range compression

To test this hypothesis on our data, for all pairs of groups in all datasets, we regressed log *FC*_*protein*_ on log *FC*_*mRNA*_ using a variant of major axis (MA) regression, and tested whether the slope is significantly different from 1 (Additional file 4: Table S3). All slopes were significantly less than 1 for the EAR and PRIMATE datasets, and for all except one pair in the MMT dataset (see Fig. 2 for examples). For the NCI60 and brain-cerebellum [MMT] the slopes were significantly higher than 1. When using ordinary least square (OLS) regression, all the slopes calculated were significantly less than 1 (*q − value* ≤ 0.01), consistent with the aforementioned range compression phenomenon (discordance between the regression methods is demonstrated in Figure S6 [Additional file 2]). However, MA regression is not sensitive to regression dilution bias, which can severely lower the estimate of the slope in OLS regression [19]. Using MA, it appears that the range compression is a common phenomenon for pairs of tissues, or species. For cell lines, an opposite phenomenon of range expansion occurs.

**Fig. 3:**
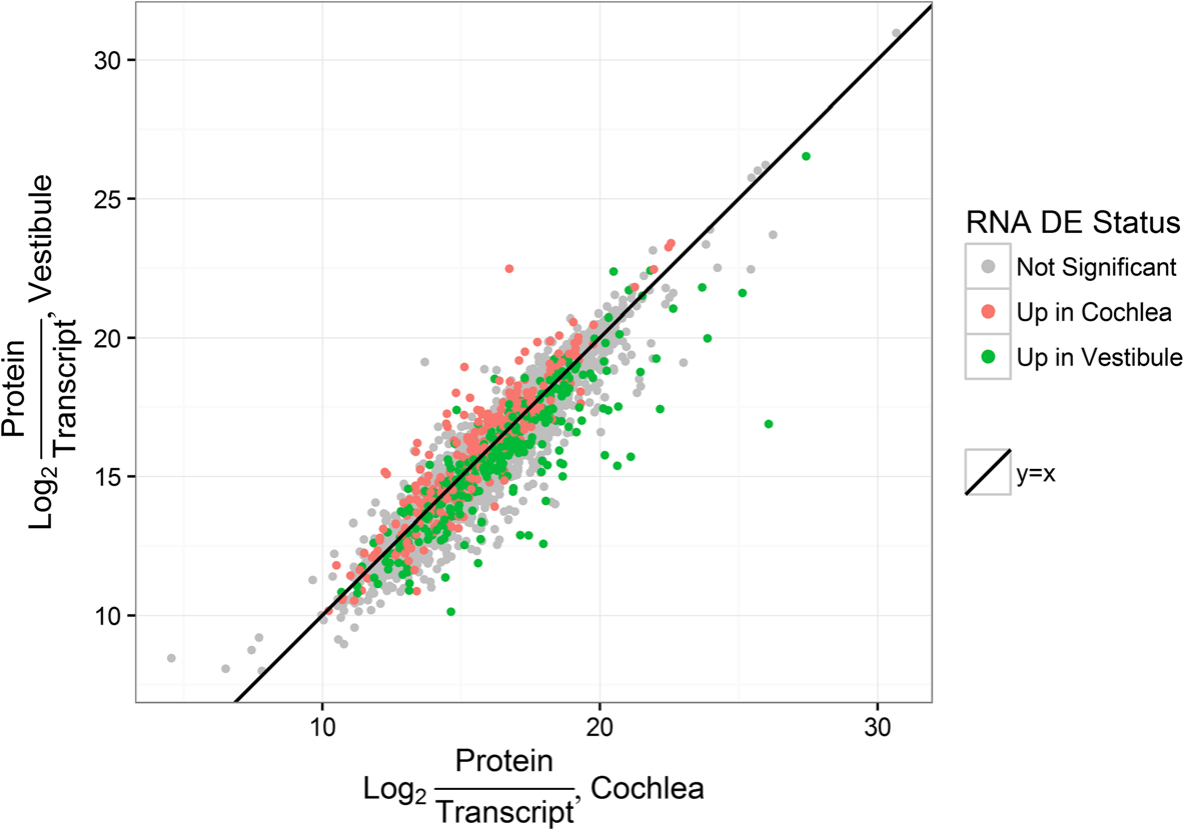
Protein-transcript ratio (PTR) and differential expression between two inner-ear tissues. The PTRs in the cochlea (*x-axis*) are plotted against the PTRs in the vestibule (*y-axis*), where the PTRs were calculated using mRNA data of samples SA623 and SA626 respectively. Marked in *red* are genes that are up-regulated in the cochlea, and in *green* are genes that are up-regulated in the vestibule (edgeR, *q − value* ≤ 0.05). Samples SA623 and SA626 were excluded from the differential expression analysis. The *black line* is *y* = *x*. There is a clear tendency for the genes that are up-regulated in the cochlea (*red points*) to have higher PTR in the vestibule (be above the *black line*), and vice versa. Note that to emphasize the DE status, significant (*colored*) genes are drawn at the front and may occlude some non-significant ones

Next, we used a nonparametric approach to test whether genes that are up-regulated in one group versus the other in the mRNA domain will show lower PTR in that same group versus the other. If this hypothesis is correct, it can explain the compressed ratios in the non-cancerous datasets. We formulated two complementary testing approaches: A global test that considers all the genes ranked by their mRNA *differential expression (DE) values*, and a local test that focuses on those that are DE. Importantly, we separated the repeats on which PTR and DE values are computed in order to avoid bias in the significance evaluation (see Additional file 2: Supplementary Methods, Figure S7). Figure 3 provides an example of the DE-PTR comparison in inner-ear tissues. The PTRs in the cochlea were plotted against the PTRs in the vestibule, with the genes DE between the tissues highlighted. We observe that genes up-regulated in one tissue tend to have higher PTRs in the other tissue. This property is tested by the local approach.

The global tests were significant for all group pairs in the EAR, MMT, and PRIMATE datasets (*q − value* ≤ 0.01, Additional file 5: Table S4). The results were in complete agreement with those of the local approach. The positive results support the buffering observation for all these datasets, and those of the local approach specifically indicate that within these datasets reduced protein expression changes have a major effect on the DE genes. For the NCI60 dataset, none of the pairs were significant, and all the correlations were very close to zero. Therefore, we cannot determine the presence of a compression or an amplification effect based on this approach. As mentioned before, the different cell lines have very similar expression profiles, and this might cause a low signal-to-noise ratio.

### Predicting protein abundance from mRNA levels

Next, we examined whether we can predict protein levels based on the mRNA data. We compared three estimators all of which are trained on a subset of each dataset, and examined their ability to predict the protein level in the rest of the samples. The first estimator was built on the average PTR (APTR); the second estimator, which is fold change based (FCB), assumes a constant compression ratio of the fold changes between protein and RNA; the third infers the protein levels from the average protein (AP) levels in other tissues. AP and APTR also have a weighted version, which gives higher weight to the tissues with higher similarity, and FCB has a relaxed version (RFCB) that allowed for protein levels to change exponentially between groups, independent of change in mRNA. This accounts for differences between groups in the activity of the translational mechanisms and in protein stability.

**Fig. 4:**
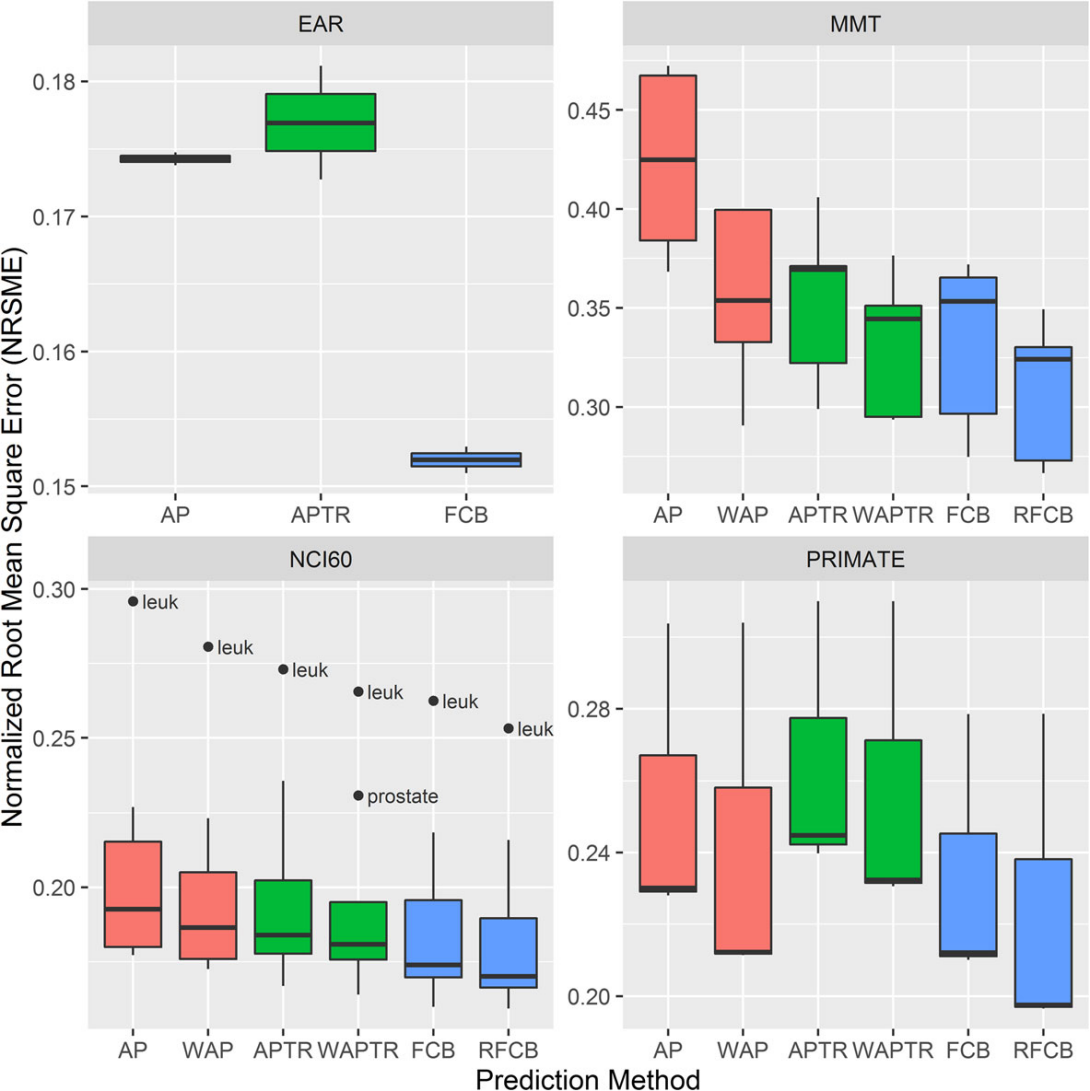
Performances of methods for protein level prediction. Boxplots show the distribution of the normalized root mean square error (NRMSE) in the prediction of protein levels, using six described methods: Averaged Protein (AP), Weighted Average Protein (WAP), Average PTR (APTR), Weighted Average PTR (WAPTR), FC Based (FCB), and Relaxed FCB (RFCB). In each tissue, RMSE values are divided by the standard deviation of the protein levels in that tissue. The error sizes are averages over tissues of 10-fold cross validation. In the EAR dataset there are only two groups, so the weighted/relaxed versions are irrelevant. Boxplots show median, a box for the middle 50% and whiskers to the largest and smallest values that are not classified as outliers. If the distance of an observation from the box is higher than 1.5 times the box size, it is classified as an outlier. Outliers are labeled

In all datasets, the FCB and RFCB models achieved better results than the others (Fig. 4). For all models, the weighted/relaxed versions achieved better results than their unweighted counterparts. The difference was very apparent for the MMT dataset, where the presence of two related tissues, brain and cerebellum, lowered the prediction error dramatically for those tissues; analysis of this dataset after the removal of one of the two still showed an advantage for the weighted versions, albeit smaller (Additional file 2: Figure S8). These findings support the use of a weighted estimator, which gives higher weights to tissues that are closer in their protein levels and PTRs.

The average improvement in the Mean Square Error (MSE) using the RFCB model over the next best weighted/relaxed model was 24.0%, 15.2%, 14.3%, 8.9% in the EAR, MMT, PRIMATE, and NCI60 datasets. Overall, the superiority of the FCB and RFCB supports the model of constant compression or expansion ratio between mRNA and protein fold-changes. Our previous analysis supports compression, at least for the EAR, MMT, and PRIMATE. The value of the compression parameter, α, of the FCB model is directly linked to the extent of compression. High variance between datasets and between groups was observed in the estimated value of this parameter (Additional file 2: Supplementary Results, Figure S9). We thus conclude that this parameter should be adjusted separately for each protein level prediction task. We also compared the protein prediction power between the different datasets, and showed that the task of predicting protein levels where one is given expression data from a similar tissue, is easier than predicting using data from less similar tissues (Additional file 2: Supplementary Results). This explains why the lowest MSE is achieved in the EAR dataset and the highest in MMT.

So far, our analysis showed the superiority of the RFCB method at the level of a dataset. This superiority still holds when moving to the level of a group, as in all groups the MSE of the RFCB predction is the lowest among all methods. Focusing on the NCI60 dataset, the greatest improvement in predictions in terms of normalized MSE is achieved for the leukemia and prostate, these cell lines having the lowest protein prediction power to begin with (Additional file 2: Supplementary Results, Figure S10). Next, we focused on the gene level, checking how well our prediction performs in predicting oncogene levels in cancer cell lines. Out of the 24 oncogenes surveyed in [20], we had full protein and mRNA data for CTNNB1, NRAS, and RB1. Using the six described methods, we predicted their protein levels in each NCI60 group, and compared the results to the measured protein levels (Additional file 2: Figure S11). For 21 out of 27 combinations of gene and group, all six predictions method performed well, with less than 2-fold difference between the expected and predicted levels. In the few cases where the difference was greater than 2-fold, the six methods were biased in their prediction in the same direction. An exception to this agreement was found in the prediction of NRAS expresion in breast and prostate cell lines, where the predictions of the AP and APTR methods suffered from ~1.4-fold prediction biases in opposite directions. In both cell lines the FCB and RFCB methods had a nearly perfect prediction.

### Differential expression indicates protein profiles are more similar than their RNA counterparts

We compared the DE genes between tissues in the EAR dataset, both at the protein and the mRNA domain. This type of comparison, as well as the comparison of the functional enrichment of the DE genes on the mRNA and protein levels, can suffer from several biases (Additional file 2: Supplementary Results). There is detection bias against lowly expressed proteins, which tend to have more missing measurements, and so our power to detect DE for a lowly expressed protein is lower. Consequently, the power to detect up-regulated functions that are performed mainly by lowly expressed proteins is lower. The problem of missing data was evident in our data for the protein domain (Additional file 2: Supplementary Results, Figure S12). To account for this effect, we reran DE using different filters on the minimum number of measurements in the protein domain. We focus here on the results when analyzing only proteins for which all measurements were available.

**Fig. 5:**
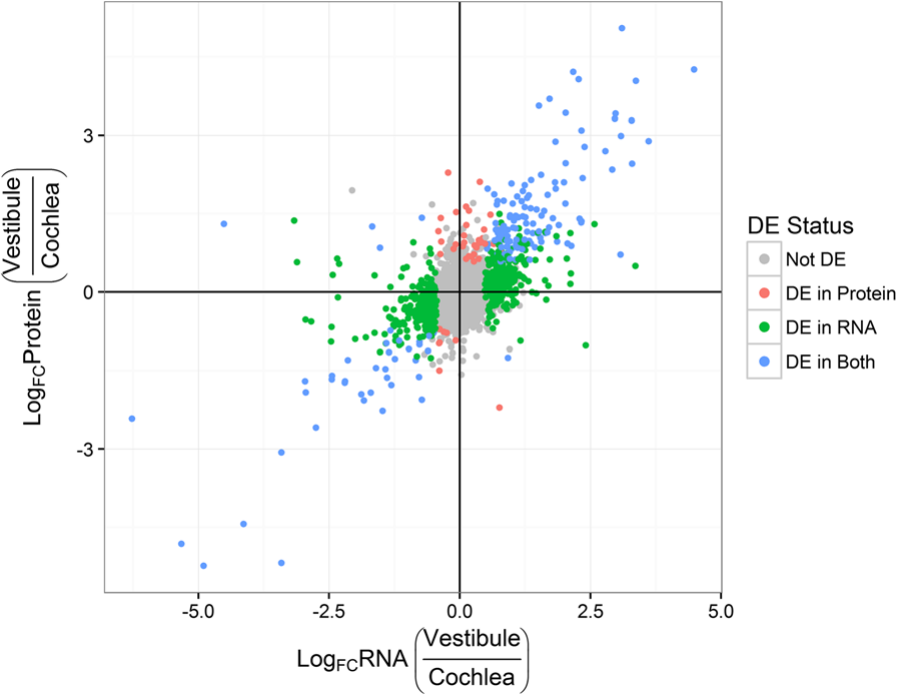
RNA and protein expression fold changes between inner ear tissues. For mRNA differential expression and fold-change estimation we used the edgeR package, with a detection threshold of *q − value* ≤ 0.05. For protein we used the samr package (two class unpaired test) with threshold *q − value* ≤ 0.1. Only proteins with measurements in all samples were included. Note that to emphasize the DE status, significant (*colored*) genes are drawn at the front and may occlude some non-significant ones

Plotting the RNA and protein fold-changes of the DE genes (Fig. 5), we observed that (i) more DE genes were found in the mRNA domain (235 versus 46 and 358 versus 156, upregulated in the cochlea and vestibule, respectively), (ii) genes found to be DE in protein were usually DE also in mRNA in the same direction (in the cochlea, of the genes upregulated in protein, 78% were upregulated in mRNA and only 2.2% were downregulated; in the vestibule, the corresponding numbers were 76 and 2.6%, respectively), and (iii) genes found to be DE in both domains had more extreme mRNA fold changes than those found to be DE only in mRNA (median FC: 2.90 versus 1.62 and 2.37 versus 1.69 for genes upregulated in the cochlea and vestibule, respectively*; q − values* = 9.4 × 10^−11^, 4.8 × 10^−20^, one-sided Wilcoxon rank sum-test). These observations imply that we expect the similarity between protein profiles to be higher than between their mRNA counterparts. We note that these results remain valid when using other filters or other DE detection procedures (Additional file 2: Supplementary Results). We could not perform this type of analysis on the MMT dataset as statistically reliable DE techniques require replicates.

### Some tissue-functionalities coded in mRNA are not manifested in protein

For mRNA and protein, we looked for GO enrichment in the set of genes up-regulated in the cochlea versus the vestibule and vice-versa (Additional file 6: Table S5). We observed that the terms found in the mRNA domain represent a far broader list of functions than those found in the protein domain, when summarizing over the enrichments found using all filters. However, when comparing only the lists of enrichment terms found in the full data filter (i.e., using only the proteins with measurements values in all samples), the lists were similar in size, yet quite distinct in content. Only three terms overlapped in the vestibule, representing 33 and 30% of the enrichments in the mRNA and protein, respectively, and none overlapped in the cochlea. The similar size of the two lists was surprising, considering the much higher number of DE genes in the mRNA domain. It was also unexpected to see so little overlap between the lists, as 77% of the genes found to be DE in protein were also DE in the same direction in mRNA in this analysis.

The analysis in the cochlea captured the functions of cell morphogenesis and nucleobase catabolic process in the mRNA domain, and the function of sensory perception in the protein domain. Importantly, the functions enriched in the protein domain were found in the mRNA domain when using less stringent filters, but not vice versa.

The analysis of the vestibule identified functions related to cell development and morphogenesis, biological adhesion, and response to wounding in both domains. Responses to general stimulus and chemicals, localization and cellular component movement, and renal system development, known to be related to ear development [21], were functions observed only in mRNA enrichments. Terms relating to anatomical structure morphogenesis, and specifically to the process of endochondral bone morphogenesis, were enriched in the protein, as was the less expected term of phagocytosis. Here also, all the functions enriched in the protein domain were either found, or similar terms to them were found, in the mRNA domain with less stringent filters. In contrast, none of the functions unique to the mRNA domain were found in the protein domain when using less stringent filters. These observations fit the hypothesis that some functionalities coded in mRNA are not manifested in protein.

An exception to this behavior, that is, a function that is relatively more ‘active’ in the protein domain, was found using a different approach for detecting post-transcriptional regulated functionalities, in which we compared the functional profiles [22] of the DE genes between protein and mRNA. Using this approach, we concluded that the function of cell adhesion is post-transcriptionally controlled in the vestibule, with a relatively large number of genes that are not DE in the mRNA, but are so in the protein (Additional file 2: Supplementary Results; Additional file 7: Table S6).

We performed enrichment analysis on the MMT dataset as well, by ranking the genes according to their fold-changes in protein and mRNA, and using a cut-off independent approach [23] to identify enrichments in both domains (Additional file 8: Table S7; see Additional file 2: Supplementary Methods). Inspired by [24], we scored each pair of tissues according to how specific the terms that arise from the enrichment analysis are, to either the protein or the mRNA domain (Additional file 2: Figure S13). For most pairs of tissues, this analysis showed that there are more functions unique to the mRNA than to the protein. This was very prominent in functions upregulated in the heart compared to the liver. In contrast, functions up-regulated in the cerebellum, compared to the liver and kidney, were more specific to the protein domain. Next, we pooled the unique terms from all pairs, to determine which functions are uniquely enriched in one of the domains. After aggregating the results at the level of ‘GO slim’ [25], we observed that protein modification and amino acid metabolism, as well as transport, including vesicle-mediated transport, tend to be unique in the protein domain (Additional file 2: Figure S14). In contrast, lipid metabolism and catabolic processes, along with stress response, are more transcriptome-specific functions. Terms related to cell death, cell adhesion, and immune system response, all appeared multiple times (≥5) and only in the mRNA comparisons.

To complete the analysis we also analyzed genes that show relatively high expression in the mRNA, but their measurements are completely missing from the protein. We performed this analysis on all datasets. For some of the cancerous cell lines, we found tumor related functionalities that are controlled through post-transcriptional repression (Additional file 2: Supplementary Results; Additional file 9: Table S8), namely, functionalities that are coded in mRNA but are less ‘active’ in protein.

## Discussion

In this study we compared mRNA and protein expression across diverse datasets: mouse inner ear tissues, mouse organs, cancer cell lines and primate lymphoblastoids. We observed that the correlations in protein expression between groups are higher than the correlations in mRNA expression, across all datasets. It was previously observed that across *taxa* protein levels are more conserved than mRNA levels [8]. We showed this phenomenon across *tissues* as well, and explained it by changes in the transcript level that are attenuated at the protein levels. A direct outcome of this phenomenon is the compression of large differences in mRNA expression to smaller ones in the protein domain. This is the first observation of this phenomenon for non-proliferating tissues, though it was previously seen in proliferative ones [7]. Moreover, the aforementioned studies used OLS regression, which is known to suffer from a strong dilution bias [12]. Using the more robust MA regression instead, we provided evidence for such compression in EAR, PRIMATE and in MMT (except for one tissue pair). In NCI60 and the brain-cerebellum pair [MMT] the regression results supported expansion, instead of compression.

When comparing tissues that are very similar in level of expression, small biases can render the regression invalid. In order to solve this issue, we tried a non-parametric approach, which can be less powerful but is not dependent on an underlying linear model. Using this approach, we showed buffering for all datasets except NCI60. We therefore conclude that a partial buffering between translation and transcription exists in the MMT, EAR, and PRIMATE datasets. For NCI60, the results were insignificant, and supported neither compression nor its opposite, signal amplification. Perhaps a more powerful test (for example, a random effects model [12]) may provide the answer. For the PRIMATE dataset such an observation was made previously [10]. In this study, by addressing some of the limitations of that statistical analysis, we reaffirmed the correctness of the observation (Additional file 2: Supplementary Results).

We did not necessarily expect to see the same phenomena in cancer cell lines as in healthy tissues, for obvious reasons: cell lines are programmed to proliferate, whereas cells in healthy tissues divide slowly, if at all; cell lines somewhat lose their resemblance to their tissue of origin, thus becoming more similar to a “global cancer pattern”; and cell lines of the same origin may diverge in their transcriptomic and proteomic profiles as they follow different paths of cancer evolution. In addition, the post-transcriptional regulation may be altered or even damaged in cancer. We showed one manifestation of these biological differences, namely the lesser ability to separate NCI60 samples based on their origin, compared to the EAR and MMT datasets. Since the cell lines are more similar to each other in their expression profiles, the compression effect is expected to be less dominant in cancer.

A translational model has been proposed, where transcriptional signals are amplified by translational regulation [12]. The existence of an amplifying mechanism might appear to contradict the buffering suggested here. However, the authors studied budding yeast, a single cell type. In this model an increase in the mRNA level of a transcript would translate into an exponential increase of the matching protein, while our analysis is based on multiple tissues. In each tissue the transcriptional, translational and post-translational regulations are fine-tuned to enable the correct function of the tissue. Both mechanisms can coexist, i.e., the expression profiles that we observed result from a balance between compressing and amplifying mechanisms. The first is related to the tissue identity (perhaps through epigenetic marks), and the second is connected to the way the translational apparatus of a cell functions. A very similar argument was made in [12], in the context of different species. We speculate that the contradicting evidence we observe for buffering in groups that are more similar to one another might be the result of such balance; i.e., in such groups, the balance between the two mechanisms leans towards amplification.

What biological mechanism explains the buffering observation? Decoupling is achieved by changing the translation rates, the protein degradation rates, or both. We cannot distinguish between these three options using our analysis, yet according to the literature, protein translation is assumed to be the major contributor to the variance of protein concentration [18], and was shown to change through tissue differentiation [5]. Hence we can speculate that the translation rate is the factor that is changing between the two tissues, although in a different context, of expression quantitative trait locis in LCLs, the buffering observed between protein and mRNA was attributed mainly to protein degradation [26]. In Supplementary Results [Additional file 2] we discuss explanations from the literature [6, 7] as to how the coordination of translation and transcription is achieved, and demonstrate that alternative polyadenylation, one of the proposed mechanisms [7], plays only a minor role, if any, in this balance in the EAR dataset.

We acknowledge the possibility that mRNA measurement error might cause an overestimation of the buffering effect. It is well known that distinct tissues may contain different amounts of RNAase that degrade mRNA at dissimilar degrees and with different specificities [27]. Given the impact mRNA integrity has on transcript quantification [28], these differences may result in measurement errors that are inconsistent between tissues. By using ribosome profiling data instead of RNA-seq measurements, one can avoid this problem altogether, and obtain more rigorous results. Another source of error is the number of amplification cycles and the precise PCR conditions used for each sample. We used the Spearman’s correction to mitigate the between-replica error but we did not account for systemic errors between tissues. Tighter experimental controls, together with more elaborate statistical normalization techniques, can address this potential error.

We demonstrated how the prediction of protein can be improved by taking the range compression into account. Models that allow PTR to vary between tissues in a direction that buffers the change in protein levels (R\FCB), performed better than models that did not allow this variation or ignored RNA levels altogether. The improvement in the prediction error was between 9 and 24%, depending on the dataset. The largest improvement was achieved in the EAR, but in this dataset the prediction was very good to begin with. In the PRIMATE dataset the smaller improvement of 14% can make a large difference in the prediction quality. This enhanced ability to predict protein levels can be utilized, for example, to better predict disease status using machine learning. The higher accuracy exhibited by the RFCB method in the prediction of the NRAS protein level in breast cancer cell lines, supports its usage in disease status evaluation, as overexpression of NRAS is associated with poor prognosis in breast cancer [29]. In the future, as understanding of mRNA-protein relationship improves, more sophisticated prediction tools can be developed that will be aware of this mechanism and explore different features of it (for example, whether it saturates in higher mRNA expression levels).

If buffering worked in the linear fashion captured by the FCB model, and the noise level was similar in the measurements of protein and mRNA, we would expect the correlations between tissue pairs in the protein and the mRNA domains to be almost equal. We observed, however, that the correlations in the protein domain were higher. This is a surprising finding, especially in light of the higher noise level in protein, suggesting that a more powerful nonlinear buffering model could be described. Another support for a stronger buffering comes from the number of DE genes we found, which was much higher in the mRNA domain. As mentioned, the protein measurements are slightly noisier, though probably not to the extent that justifies these high differences.

In the enrichment analysis we observed that the functionalities represented at the protein domain were, by and large, a subset of the functionalities represented at the mRNA domain, which were far more numerous. The fact that we find less enrichment categories in protein is partially explained by the missingness pattern in the protein measurements: we have less chance to detect categories in which some or all of the genes are lowly expressed in the protein domain (or characterized by low detectability by MS). Focusing on the subset of genes with full measurements in protein allows a more fair comparison, but nearly ignores the possible differences between those ‘low expression’ categories. In that comparison we found a similar number of enrichment categories for protein and mRNA. The lists differ greatly; however, we notice that the categories that were found in the protein and not in the mRNA, were represented in the analysis of the full, non-filtered, mRNA data. We can conclude that all the functionalities that are represented in the protein are also evident in the mRNA data. For the opposite direction it is much harder to tell; to accurately answer this question we need to somehow predict the missing values in the protein, or develop an enrichment analysis tool that is aware of the ‘missing not at random’ nature of the data [30].

Why does one tissue maintain higher mRNA levels but the same protein levels compared to another, where such practice requires more energy from the cell? We suggest that functionally distinct tissues possess different mRNA profiles but similar protein profiles, in rest, as part of a preparation for a stimulus. Under some stimulus a translational inhibition is removed from a gene (or group of genes) that is DE between the tissues only at the mRNA domain, so that the tissue that possesses higher levels of the gene’s transcript will synthesize the protein faster. Indeed, one of the virtues attributed to translational control is the possibility of rapid response to external stimuli [31]. Moreover, when exposing mammalian cells to stress induced by dithiothreitol, mRNA- and protein-level regulation contribute equally to the change in protein expression [32], demonstrating the importance of protein-level regulation under stress. If our suggestion is correct, it might be beneficial to measure both mRNA and protein levels in order to deduce functionality of genes. If a gene is DE at the protein domain, then the protein is important to the function of the resting tissue. If a gene is DE only at the mRNA domain, then it is required for the tissue functionality under some stimulus.

The fact that the vestibular up-regulated genes are enriched for response to stimulus and chemicals only in the mRNA domain might be a manifestation of this hypothesis, as a role for these responses in the normal development of the ear is not known. Also fitting this hypothesis are the multiple immune related terms found in the mRNA domain, in the analysis of the non-filtered data. Nevertheless, the lack of these terms from the protein analysis might be related to a relatively low expression of the genes in these categories. In the MMT analysis we see a similar pattern. Response to stress terms are enriched in mRNA data and not in protein, and those of immune system response are unique only to mRNA. In the literature we can find examples where the translational regulation of genes changes in response to heat shock [33], hypoxic stress [34], changes in iron concentration [35], and exposure to EGF [24]. It is interesting to explore whether the genes activated in these responses are highly expressed in the mRNA domain, compared to a tissue that is not normally subjected to these types of stress, even before the actual exposure.

## Conclusions

Our work demonstrates that protein levels are more conserved between tissues than mRNA levels. We employed this observation to improve the prediction of protein levels in a non-proliferating tissue based on the mRNA levels measured in that tissue, by using data from several other tissues. A biological explanation is proposed as to why tissues maintain different levels of mRNA and similar levels of protein, by providing examples where this phenomenom serves as a preparation for a stimulus.

## Methods

### EAR mRNA data generation

Cochlear and vestibular sensory epithelia were dissected from 20 inner ears of 10 P0 C57Bl/6 J mice, generating 2.4 and 1.5 μg of total RNA, respectively. Four hundred and fifty nanogram RNA from each sample was used to create libraries with the TruSeq Stranded mRNA Sample Prep Kit (Illumina), followed by high-throughput sequencing at 100 bp paired end (PE) at the Technion Genome Center, Haifa, Israel. Six samples were generated, three cochlear and three vestibular, for sequencing in triplicate. Read quality was assessed using ShortRead and reads were aligned using tophat2 against a mouse reference genome (Mus_musculus.GRCm38.74). BAM files were manipulated using Samtools and per-gene counts of the reads were computed using htseq-count. edgeR was used for calculating DE, fold changes and RPKM normalized values. Only genes that have one read per million in three or more of the samples were included in the analysis. See [36] for references to each software tool.

### EAR proteomics data generation

Cochlear and vestibular sensory epithelia were dissected from 15 P0 C57Bl/6 J mice, with samples from each set of five mice pooled to generate one of three replicates of protein from cochlear or vestibular tissues. Protein samples were reduced with DTT and alkylated with iodoacetamide followed by in-solution digestion with trypsin. Peptides from two replicates were analyzed by single LC-MS runs and one replicate was further separated into six fractions, each analyzed by LC-MS on the EASY-nLC1000 UHPLC coupled to the Q-Exactive MS. Raw MS files were analyzed with MaxQuant and the Andromeda search engine. The label-free algorithm was used for protein quantification with a minimum two ratio counts for normalization. The database search was performed against the Mouse Uniprot database (2013) with 50,807 entries and a list of common contaminants. False discovery rate (FDR) was determined using the forward-reverse approach, and set to 1% FDR on the peptide and protein levels. Database search parameters included Trypsin/P as the proteolytic enzyme, N-terminal acetylation and methionine oxidation as variable modifications, and carbmidomethyl cysteine as a fixed modification. Maximum two miscleavages and a maximum peptide charge of +7 were allowed. First database search was used for mass recalibration with an error tolerance of 20 ppm followed by the main Andromeda search with mass tolerance of 4.5 ppm for MS spectra and 20 ppm for the MS/MS spectra. Peptide length was set to a minimum of seven amino acids. Analysis of the raw MS data identified 7244 proteins, with correlations of 0.9 and 0.95 between biological replicates of cochlea and vestibule, respectively. The expression profiles are available in Additional file 1: Table S1. The mass spectrometry proteomics data have been deposited to the ProteomeXchange Consortium [37] via the PRIDE partner repository with the dataset identifier PXD003379.

### MMT RNA data preprocessing

Multiple Mouse Tissues (MMT) data were downloaded as fastq files from ArrayExpress database (www.ebi.ac.uk/arrayexpress) under accession number E-GEOD-30352 and processed into read counts using the same protocol and reference genome as the EAR data. Out of 36,441 genes, only 16,969 genes that have one read per million in three or more of the samples were included in the analysis. We used samples for both wild mice and C57Bl/6 J mice. There was clear separation of the samples by tissue and only poor separation by strain (Additional file 2: Figure S15). Therefore, we chose to summarize tissue information from both strains in order to increase the statistical power.

### MMT protein data preprocessing

Proteomic data was taken from [15]. For each tissue, the study provides two types of measurements, the MS intensity of the light version of the peptide, and the intensity ratio of heavy and light versions of the peptide. The choice of which quantity to use in each analysis is detailed in section ‘Units of measurements’.

Protein samples of three different brain regions were merged into a single summary sample by computing a weighted mean. This summary sample can be compared to the RNA brain samples that were produced from entire brain except olfactory bulb and cerebellum [14]. The weights used, based on the volume proportions of the regions in an adult C57BL/6 J mouse brain [38], were 61.9, 24.3, and 13.8% for the cortex, medulla and midbrain respectively. The midbrain volume is computed from the sum of volumes of the superior and inferior colliculi, central gray, and the structure named “the rest of midbrain”. Similarly, protein samples of two different kidney regions were merged into a single representing sample. The weights used here were volume proportions of the regions in a newborn Swiss Webster mouse [39] (58.5 and 41.5% for the cortex and medulla, respectively).

### NCI60 RNA data retrieval

Transcriptomics (series accession GSE32474 [16, 40]) and proteomics [17] data were downloaded from: http://129.187.44.58:7070/NCI60/.

### Units of measurement

In the MMT and PRIMATE datasets proteins were quantified using the SILAC technique, which gives for each protein the ratio of expression between an individual sample to an internal standard (SILAC tissue). In both datasets, we also quantified the protein levels based on the intensity of the peptides in the light version, which corresponds to peptides from the non-SILAC tissue. The absolute levels were used in the production of summary statistics, calculation of correlations, and prediction of protein levels, whereas the SILAC ratios were used in MDS plotting, DE analysis, and testing whether PTRs vary in a direction that reduces protein divergence. The use of SILAC ratios was preferred in the last scenarios as it yields a more accurate estimate of protein abundance between two proteomes [41].

#### EAR

The protein unit is *LFQ Intensity*/*MW*, where LFQ is a commonly used normalization for protein intensity [42], and MW is the molecular weight in kDa. The mRNA unit is RPKM (Reads Per Kilobase per Million mapped reads) [43]. For DE analysis using edgeR [44], the read counts were used.

#### MMT

The unit used for absolute protein levels is *Intensity. L*/*MW*, where Intensity.L is the sum of the measured intensities of the light version of the peptides composing the protein. The unit used for relative protein levels is Ratio.H.L.normalized, where Ratio.H.L.normalized is the ratio of the heavy to light intensities, after applying normalization as in [15]. A mix of SILAC mouse tissues served as an internal standard. The mRNA unit is RPKM. For DE analysis using edgeR, the read counts were used.

#### NCI60

The protein unit is *LFQ Intensity*/*MW*. The mRNA unit is the intensity level measured from the microarray chip, normalized as in [16].

#### PRIMATE

The unit used for absolute protein levels is iBAQ [18], based on the intensities of the light version of the peptides composing the protein. The unit used for absolute mRNA levels is RPKM. The unit used for relative protein levels is Ratio.H.L.normalized. A single human SILAC served as an internal standard. The unit used for relative mRNA levels is *RPKM*_*sample*_/*RPKM*_*standard*_, using the same reference cell line. The relative mRNA levels were used for the same purposes as the relative protein levels.

### Spearman’s correction

When we wish to compute the correlation between two parameters, measurement errors of each parameter weaken our results. Spearman’s correction accounts for this effect and utilizes repeated measurements to correct it. We can infer the Pearson correlation between the latent variables φ and ψ, given N measurements of φ, marked *x*_1_, …, *x*_*N*_, and M measurements of ψ, marked *y*_1_, …, *y*_*M*_. The following estimator for the Pearson correlation between φ and ψ is then used [12]:

$$ {\widehat{r}}_{\phi \psi}=\frac{{\left({\displaystyle {\sum}_{i, j}^{N, M}{r}_{x_i,{y}_j}}\right)}^{\frac{1}{N\times M}}}{{\left({\displaystyle {\sum}_{i< i\hbox{'}}^N{r}_{x_i,{x}_{i\hbox{'}}}}\right)}^{\frac{1}{N\left( N-1\right)}}{\left({\displaystyle {\sum}_{j< j\hbox{'}}^M{r}_{y_j,{y}_{j\hbox{'}}}}\right)}^{\frac{1}{M\left( M-1\right)}}} $$

Where ${r}_{x_i,{y}_j}$ is the empirical correlation between measurements *x*_*i*_ and *y*_*j*_. We assume that all the empirical correlations are positive. The estimator is in [0,∞).

To correct the mRNA correlation between the groups, we treat φ as the levels of mRNA in one group, and ψ as the levels in the other group. We do the same for protein levels. Note that this method can also be used to correct mRNA-protein correlations within a group, treating φ as the levels of mRNA, and ψ as the levels of protein in that group.

### MDS plots

Multi-dimensional scaling was used to plot and visualize sample similarity. Plots were calculated using the function cmdscale in package stats (https://www.r-project.org/). For the MMT dataset, the relative protein levels were used.

### Regressing log ***FC***_*protein*_ on log ***FC***_*mRNA*_

For all pairs of groups in all datasets, we regressed log ***FC***_*protein*_ on log ***FC***_*mRNA*_ using ordinary least square (OLS) or a variant of the major axis (MA) regression. For EAR, MMT, and PRIMATE we used regular MA. For NCI60 we used scaled MA (SMA). The choice of which variant of MA to use followed [45] (see Additional file 2: Supplementary Methods). We employed three different versions of F − test supplied in the smatr package [46] to test whether the slope is significantly different from 1 for OLS and (S)MA regression. We applied FDR correction for each dataset and method separately.

### Protein levels prediction models

Assuming we have *T* − 1 groups with matching mRNA and protein profiles, and we want to predict the protein levels in a new group *T*, using the data from the first *T* − 1 groups and the mRNA levels in group *T*.

We compared three different estimators:

1. **Average PTR (APTR):** It was previously suggested to use the average translational efficiencies measured in the first *T* − 1 groups, and multiply them by the matching mRNA levels in group *T* [8]. A trivial linear model describing this prediction for a single gene is:

$$ \log {P}_T=\frac{1}{T-1}{\displaystyle \sum_{i=1}^{T-1} \log \frac{P_i}{R_i}{R}_T} $$

Where *P*_*i*_ and *R*_*i*_ are the measured protein and mRNA levels, respectively, in group *i*. This model can generalized by giving weights to the different groups. The result is called **Weighted Average PTR (WAPTR)** estimator. Weights are obtained by regression.

2. **FC Based (FCB):** A different model assumes linear relationship between log*P* and log*R* (similar to [7]). If for group *i* log *P*_*i*_ = *α* log *R*_*i*_ + *β*, then for two groups: $\log \frac{P_1}{P_2}=\alpha \log \frac{R_1}{R_2}$. α is estimated by regression. We expect 0 < α < 1, in concordance with our previous results. By averaging over all groups, we obtain the following estimator for log *P*_*T*_:

$$ \log {P}_T=\frac{1}{T-1}{\displaystyle \sum_{i=1}^{T-1}\left(\alpha \log \frac{R_T}{R_i}+ \log {P}_i\right)} $$

Or in a different form, which shows the relation to the APTR estimator:

$$ { \log {P}_T}_{FCB}={ \log {P}_T}_{APTR}+\frac{1}{T-1}{\displaystyle \sum_{i=1}^{T-1}\left(1-\alpha \right) \log \frac{R_i}{R_T}} $$

To generalize the model by allowing group weights, the simplest way assumes an exponential scaling of the protein levels between different groups, that is *γ*_*i*_ log *P*_*i*_ = *α* log *R*_*i*_ + *β*, with *γ*_*T*_ = 1. This would yield the **Relaxed FCB (RFCB)** estimator:

$$ \log {P}_T=\frac{1}{T-1}{\displaystyle \sum_{i=1}^{T-1}\left(\alpha \log \frac{R_T}{R_i}+{\gamma}_i \log {P}_i\right)} $$

The group-specific exponents are obtained by regression.

3. **Average Protein (AP):** The simplest estimator is averaging over the protein levels in the other groups, ignoring the mRNA data:

$$ \log {P}_T=\frac{1}{T-1}{\displaystyle \sum_{i=1}^{T-1} \log P} $$

This model can also be expanded to give weights for the different groups (**Weighted Average Protein (WAP)** estimator). Weights are obtained by regression.

### Scoring prediction models

For each dataset we included only the genes for which we had proteomic and transcriptomic data from each of the groups, i.e. a measurement was available for at least one sample belonging to the group (5048, 3514, 3223, and 3394 genes in EAR, MMT, NCI60, and PRIMATE datasets, respectively). We then averaged the data over the repeats in each group. We iterated over the groups, each time setting another one as missing. For each of the aforementioned models we fitted a regression model that allowed scaling of the original estimator and also included an intercept. We performed 10-fold cross-validation on the fitted model, and collected the Root Mean Square Error (**RMSE**), using the DAAG package (cran.r-project.org/web/packages/DAAG). For each group we divided the RMSE by the standard deviation of the protein levels in the group. The result is a dimensionless measure for prediction quality called **NRMSE**, which can be used to compare predictions across datasets.

We followed a different procedure when calculating how much of the variance in protein level is explained by a specific model. We fitted the model for each group separately, and took the median percentage of variance explained. A similar technique [47], which is more appropriate for the evaluation of prediction under a cross-validation scenario, gave results within a range of <1% of the reported results.

For the prediction of protein levels of oncogenes in the NCI60 dataset, we fitted the regression models using data from all genes except the selected oncogenes.

## Additional files

Additional file 1: Table S1. Protein Data for EAR. Results from mass spectrometry and metadata used to normalize protein intensities and to connect proteins with genes. (XLSX 1184 kb)

Additional file 2: Supplementary Data. Supplementary table legends, figures, methods and results. (PDF 2108 kb)

Additional file 3: Table S2. Summary Statistics for Datasets. Statistics are available at the level of a dataset, a group within a dataset, and a sample, for samples quantified for both RNA and protein. All statistics are based on genes with some measurements in both protein and RNA. (XLSX 46 kb)

Additional file 4: Table S3. Log FC Regression. Results of OLS and (S)MA regression of log*FC*_*protien*_ on log*FC*_*mRNA*_. (XLSX 17 kb)

Additional file 5: Table S4. Non Parametric Tests for the Relation of PTR and DE between Pairs of Groups. Results of the nonparamteric approach in demonstrating relation of PTR and DE between pairs of groups, using either *global* or *local* testing procedures. (XLSX 29 kb)

Additional file 6: Table S5. EAR Differential Expression and Enrichment. Statistics of the differential expression analysis in the EAR dataset, and emerging terms in the enrichment analysis. (XLSX 87 kb)

Additional file 7: Table S6. Cell Adhesion Annotated Genes. List of genes up-regulated in the vestibule [EAR] in either protein or mRNA, and annotated for the GO term ‘cell adhesion - GO:0007155’. (XLSX 17 kb)

Additional file 8: Table S7. MMT Enrichments and Domain Specificity. Emerging terms in the enrichment analysis of the MMT dataset, and scoring of their specificity, to either the protein or the mRNA domain. (XLSX 1597 kb)

Additional file 9: Table S8. Post-Transcriptional Repression. Statistics of the post-transcriptionally repressed genes analysis, and emerging terms in their enrichment analysis. (XLSX 63 kb)

## Abbreviations

(R)FCB: (Relaxed) FC Based
(R)MSE: (Root) mean square error
(W)AP: (Weighted) average protein
(W)APTR: (Weighted) average protein-transcript ratio
DE: Differentially expressed
FC: Fold change
FDR: False discovery rate
GO: Gene ontology
LCL: Lymphoblastoid cell line
MA: Major axis
MDS: Multi-dimensional scaling
MMT: Multiple mouse tissues
mRNA: messenger RNA
MS: Mass spectrometry
OLS: Ordinary least square
P0: Post-natal day 0
PTR: Protein-transcript ratio
RPKM: Reads per kilobase per million
SILAC: Stable isotope labeling with amino acids in cell culture
