# Supplementary material for: Reduced changes in protein compared to mRNA levels across non-proliferating tissues
Source: BMC Genomics. 2017 Apr 18;18:305. doi: 10.1186/s12864-017-3683-9 (PMC5395847; doi:10.1186/s12864-017-3683-9)
Supplement: Supplementary file 2 — Supplementary Data. Supplementary table legends, figures, methods and results. (PDF 2108 kb) [file 12864_2017_3683_MOESM2_ESM.pdf]

# Reduced changes in protein compared to mRNA levels across non-proliferating tissues

Kobi Perl, Kathy Ushakov, Yair Pozniak, Ofer Yizhar-Barnea, Yoni Bhonker, Shaked Shivatzki, Tamar Geiger, Karen B. Avraham and Ron Shamir

**Supplementary Data**

---

**CONTENTS**

SUPPLEMENTARY TABLES LEGENDS ........ 4

SUPPLEMENTARY FIGURES ........ 7

Figure S1. ........ 7

Figure S2. ........ 8

Figure S3. ........ 9

Figure S4. ........ 10

Figure S5. ........ 11

Figure S6. ........ 12

Figure S7. ........ 13

Figure S8. ........ 14

Figure S9. ........ 15

Figure S10. ........ 16

Figure S11. ........ 17

Figure S12. ........ 18

Figure S13. ........ 19

Figure S14. ........ 20

Figure S15. ........ 21

SUPPLEMENTARY METHODS ........ 22

Choice of variant of major axis ........ 22

Non-parametric approach ........ 23
EAR enrichment analysis ........ 24
EAR GOProfile analysis ........ 24
MMT enrichment analysis ........ 24
Choice of enrichment analysis tool ........ 25
EAR ID conversion and annotation mapping ........ 26
Summary Statistics ........ 26
Plotting ........ 27
Alternative polyadenylation ........ 27
SUPPLEMENTARY RESULTS ........ 28
EAR RNA-seq consistency and accuracy ........ 28
EAR MS consistency and accuracy ........ 28
Comparison of protocols used to collect mRNA and protein data ........ 29
Higher correlation between replicates in the mRNA domain ........ 29
MMT mRNA correlations are probably higher than protein correlations ........ 30
Comparing major axis and ordinary least square regression ........ 30
FCB compression parameter varies between groups ........ 31
Protein expression prediction power in different datasets ........ 31
Cell lines with lower protein expression prediction power ........ 31
Missing protein measurements makes it more difficult to compare data between mRNA and protein .. 32
EAR low abundance proteins have less chance of being observed ........ 32
Comparing differential expression results using samr ........ 32
EAR enrichment analysis ........ 33

GO profiles comparison in EAR ........ 35

Protein detection bias leads to false-classification of processes as post- transcriptionally repressed... 37

Post-transcriptional repression in EAR and MMT datasets ........ 37

Reaffirming conclusions of PRIMATE study ........ 38

Possible explanations to how coordination of translation and transcription is achieved ........ 38

Alternative polyadenylation fails to explain protein fold changes........ 39

REFERENCES........ 41

## SUPPLEMENTARY TABLES LEGENDS

**Table S1.** Protein Data for EAR. Sheets: Mass.Spectrometry – Results from mass spectrometry; Proteins.Metadata – Metadata used to normalize protein intensities and to connect proteins with genes; Common columns: Majority protein IDs - uniprot identifiers; 'Mass.Spectrometry' columns: Gene names - MGI symbols; Protein names; Protein IDs - uniprot identifiers; LFQ intensity C15, C55, C85 - LFQ normalized intensities of the gene in the three cochlear samples; LFQ intensity V15, V55, V85 - same for vestibule; PEP - Posterior error probability score; Intensity - MaxQuant raw intensity; 'Proteins.Metadata' columns: MW - Molecular weight in Dalton; ENSG - Associated Ensembl gene.

**Table S2:** Summary Statistics for Datasets. Statistics are available at the level of a dataset, a group within a dataset, and a sample, for samples quantified for both RNA and protein. All statistics are based on genes with some measurements in both protein and RNA. Sheets: Datasets, Groups, Paired.Samples – The different levels at which we calculated the summary statistics; Columns: Dataset, Group, Sample – Identifiers; N - Number of genes that have some measurement in both protein and RNA; Correlation – Correlation between protein and RNA expression levels; Unit – Unit of measurement of RNA and protein; Mean, Min, Max, IQR (interquantile range), SD (standard deviation) – Summary statistics for RNA and protein.

**Table S3.** Log FC Regression. Dataset, Group1, Group2 - group pair identifier; n - number of genes for which we had at least one measurement in both groups, in both domains; r^2 - Pearson's correlation between mRNA FCs and protein FCs; OLS.slope, OLS.p, OLS.q - slope of OLS regression, p- and q-values of the regression; (S)MA.slope, (S)MA.p, (S)MA.q - same for (S)MA regression; MA.variant – the major axis variant used, either MA or SMA.

**Table S4.** Non Parametric Tests for the Relation of PTR and DE between Pairs of Groups. Sheets: Spearman - the Spearman's rank correlation between the $\log FC_{PTR}$ and the $\log FC_{mRNA}$ vectors (*global* approach); DE_FDR_0.05 and DE_FDR_0.10 - the results of the Wilcoxon test using either FDR thresholds to declare DE genes (*local* approach); Common columns: Dataset, Group1, Group2 - group pair identifier; N - number of genes for which we could calculate both $\log FC_{PTR}$ and the $\log FC_{mRNA}$ ; 'Spearman' columns: spearman.rho, spearman.p, spearman.q - Spearman's correlation, p- and q-values of the significance test; 'DE_FDR_x' columns: n.up, n.lo - number of genes detected as DE in each direction; logFC(PTR).median.up, logFC(PTR).median.lo - The median $\log FC_{PTR}$ of the genes DE in each direction; wilcox.p, wilcox.q - p- and q-values of the Wilcoxon test. All numeric measures are medians over the sample selection scheme.

**Table S5.** EAR Differential Expression and Enrichment. Sheets: Enrichments – Enrichments of DE genes; Metadata – Number of emerging DE genes and GO terms when using different filters; DE.Statistics - Statistics of the differential expression analysis; Common column: Filter - the filter applied on the genes before the DE analysis (see Supplementary Results for filter names); 'Enrichments' columns: Domain - mRNA / protein; Group - Cochlea / Vestibule; Enriched with - GO term; #genes - number of genes annotated with the term; Raw p-value, Corrected p-value - p-value was corrected within a domain and a filter; Frequency in set (%) - frequency of genes annotated with the term in background; Gene List - the genes annotated for the GO term; 'Metadata' columns: Genes - Number of genes passing the filter; Up c, Up v - Number of genes up-regulated in the cochlea and vestibule respectively; GO c, GO v - Number of GO terms found in enrichment analysis; NT - Only for protein data, the number of genes not tested for differential expression by SAM; 'DE Statistics' columns – Up c, Up v, NT – same definitions as in 'Metadata'; logFC - Log fold-change (cochlea / vestibule); % Up in c agree w other domain, % Up in v agree w other domain - Percent of genes up-regulated in the cochlea ( \ vestibule) in the specific domain (mRNA \ protein) that are also up-regulated in the cochlea ( \ vestibule) in the other domain; % Up in c+Up in v in other domain, % Up in v+Up in c in other domain - Percent of genes up-regulated in the cochlea ( \ vestibule) in the specific domain (mRNA \ protein) that are DE in the opposite direction in the other domain; Higher FC when up in c agree w other domain, Lower FC when up in v agree w other domain - P-values of testing whether fold-changes are more extreme when the gene is DE in both domains than when it is DE in one domain (mRNA \ protein); One-sided Wilcoxon rank sum-test; We use mRNA FCs and protein FCs for the mRNA and protein domains respectively.

**Table S6.** Cell Adhesion Annotated Genes. List of genes up-regulated in the vestibule [EAR] in either protein or mRNA, and annotated for the GO term 'cell adhesion - GO:0007155'. Columns: Entrez, Symbol - identifiers; DE in mRNA, DE in protein - whether the genes were DE in either domains.

**Table S7.** MMT Enrichments and Domain Specificity. Sheets: MMT.Enrichments - Enrichments in the MMT dataset for each pair of groups being compared (q-value <= 0.1, GOrilla); Pair.Specificity - For each pair of groups, the specificity of terms to either the protein or the mRNA domain; Slim.Specificity - For each GO slim term, the specificity of the descendant terms to the protein or the mRNA domain; Common columns - Group.Up,Group.Down (MMT.Enrichments, Pair.Specificity) - The groups being compared; GO.Term \ GO.slim, Description (MMT.Enrichments, Pair.Specificity) – Relevant GO term and its description; mRNA.Specificity, Protein.Specificity (Pair.Specificity, Slim.Specificity) – Specificity of the GO terms to the mRNA \ protein domain after aggregating by the groups being compared, or by an ancestor GO slim term (0-1, 1 is most specific); Combined.Specificity (Pair.Specificity, Slim.Specificity) - Protein.Specificity minus mRNA.Specificity; 'MMT.Enrichments' columns: Domain - mRNA / protein; p.value, q.value - p-value and q-value for the enrichment. q-values provide correction for multiple testing within a pair of groups and a direction; Enrichment,N,B,n.small,b.small – GOrilla's parameters of

enrichment, detailed in the 'README' sheet; Genes - The list of associated genes that appear in the optimal top of the list; Max.Similarity - The maximal similarity (0-1) of the GO term to a term in the equivalent list in the other domain; 'Slim.Specificity' columns - N.Terms.Unique, N.Terms.Total - Number of unique \ non-unique GO terms that were aggregated into the GO slim; p.value, q.value - P-value and q-value for testing whether the proportion of unique mRNA terms out off all unique terms equals p (p=0.56, two-sided proportion test).

**Table S8.** Post-Transcriptional Repression. Sheets: Enrichments - enrichments of the post-transcriptionally repressed genes; Metadata - metadata about the number of genes post-transcriptionally repressed; Common columns: Dataset, Group - group identifier. ’Enrichments’ columns: Enriched with - GO term; #genes - number of genes annotated with the term; Raw p-value, Corrected p-value - p-value was corrected within a dataset and a group; Frequency in set (%) - frequency of genes annotated with the term in background; Gene List - the genes annotated for the GO term. ’Metadata’ columns - Detected - number of genes for which both mRNA and protein were detected; Not detected - Number of genes for which only mRNA was detected; Above Threshold - number of genes above the ’detectability’ expression threshold; Post-Transcriptionally Repressed - number of genes that are post-transcriptionally repressed out of those above the expression threshold.

## SUPPLEMENTARY FIGURES

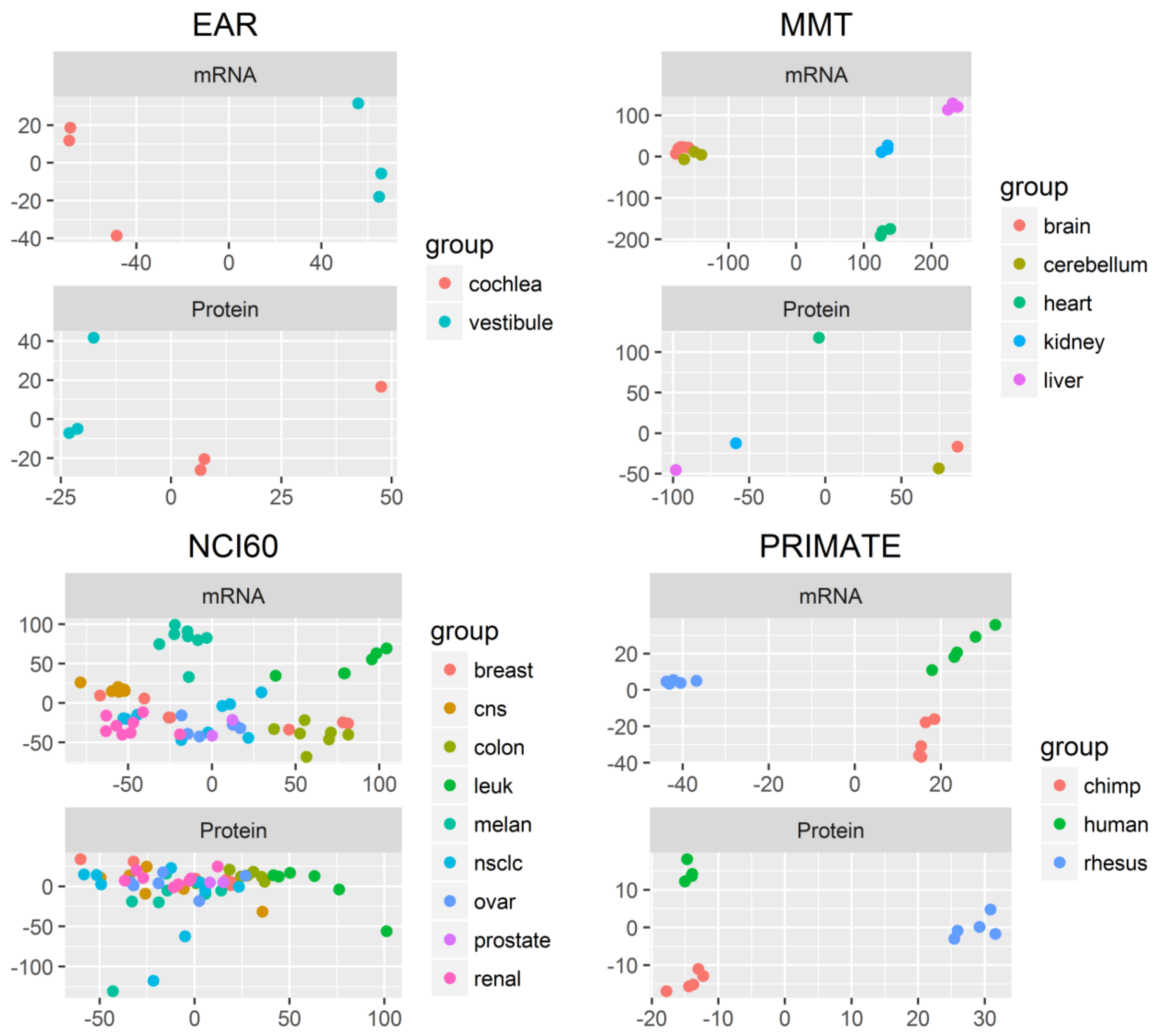


**Figure S1.** MDS plots comparing samples in the different datasets according to their mRNA or protein expression. The upper and lower figures for each dataset are the MDS plots according to the mRNA or protein expression, respectively. The x- and y-axis are the first and second coordinates, respectively. The samples are colored by their group. The groups are clearly separable by both mRNA and protein, in all but the NCI60 dataset, where the separation becomes less clear. The MDS plots are based on all the data, and not just the portion of genes for which we find expression in both domains.

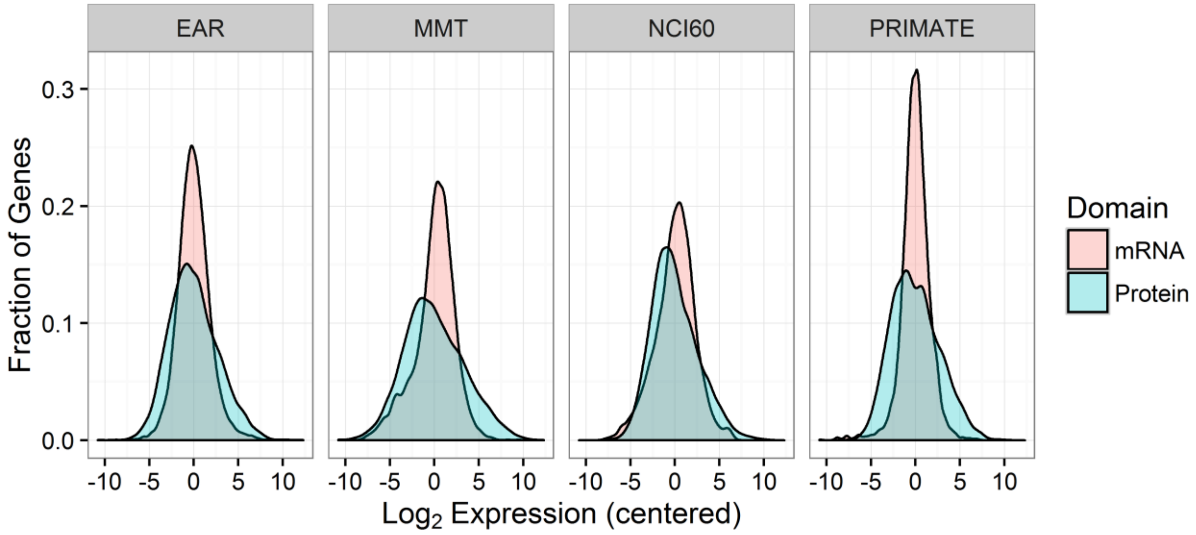


**Figure S2.** Dynamic range of expression in mRNA and in protein. The absolute levels of expression in mRNA (red) and protein (blue) are displayed in a density plot. The levels were centered around 0 to allow comparison of the dynamic range between mRNA and protein. For measures of variability (standard deviations and interquantile ranges), see Table S2 [Additional file 3].

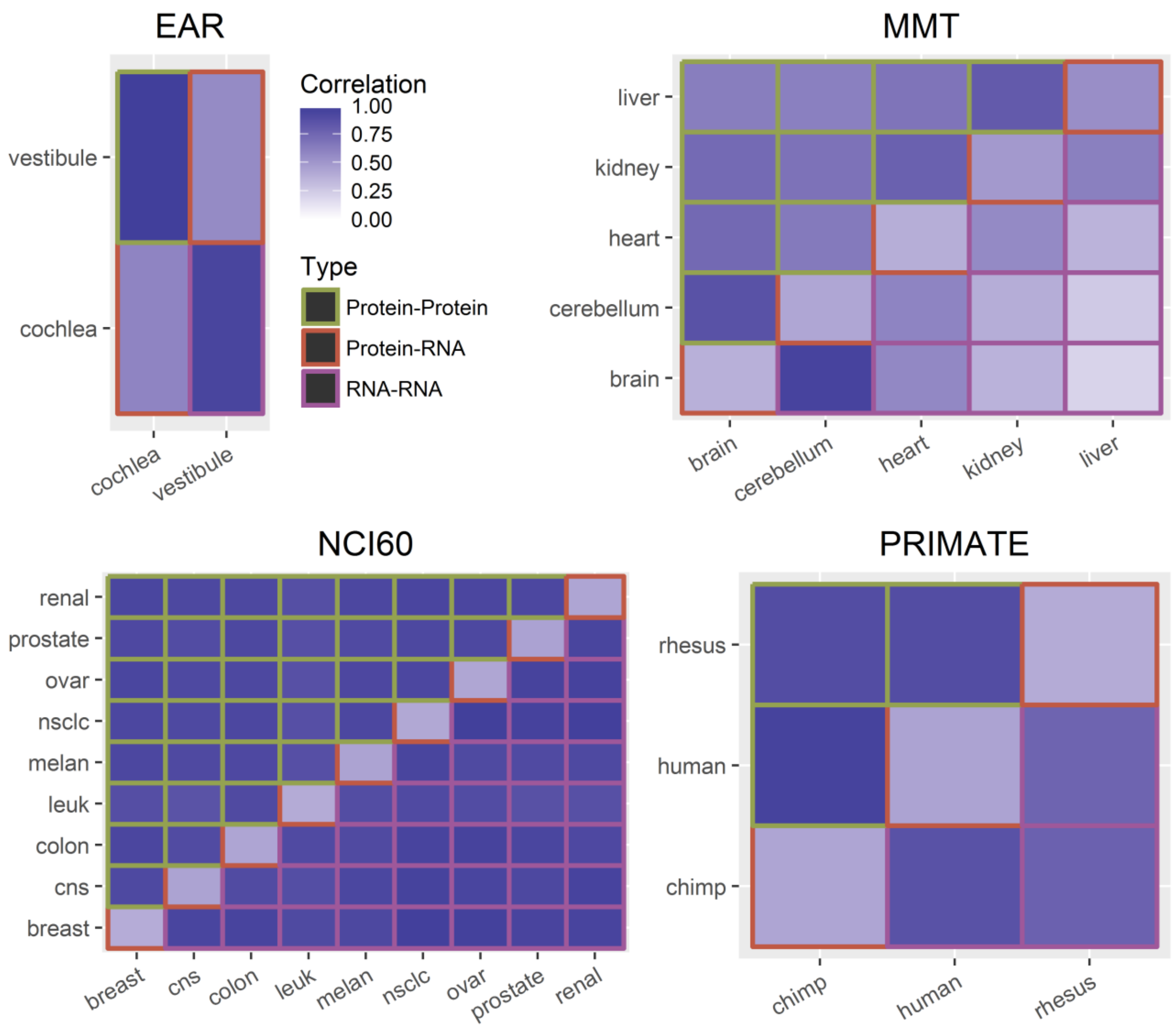


**Figure S3.** Protein and mRNA correlations between groups for different datasets. Each subfigure describes the Pearson's correlation (r) between expression levels in one dataset. The upper and lower triangles contain the protein-protein and mRNA-mRNA correlations between pairs of groups, respectively. The diagonal contains the protein-mRNA correlations within each group. Darker color corresponds to higher correlation. The correlations are not Spearman corrected because the correction cannot be applied on the MMT dataset, and this figure is intended for comparison between datasets.

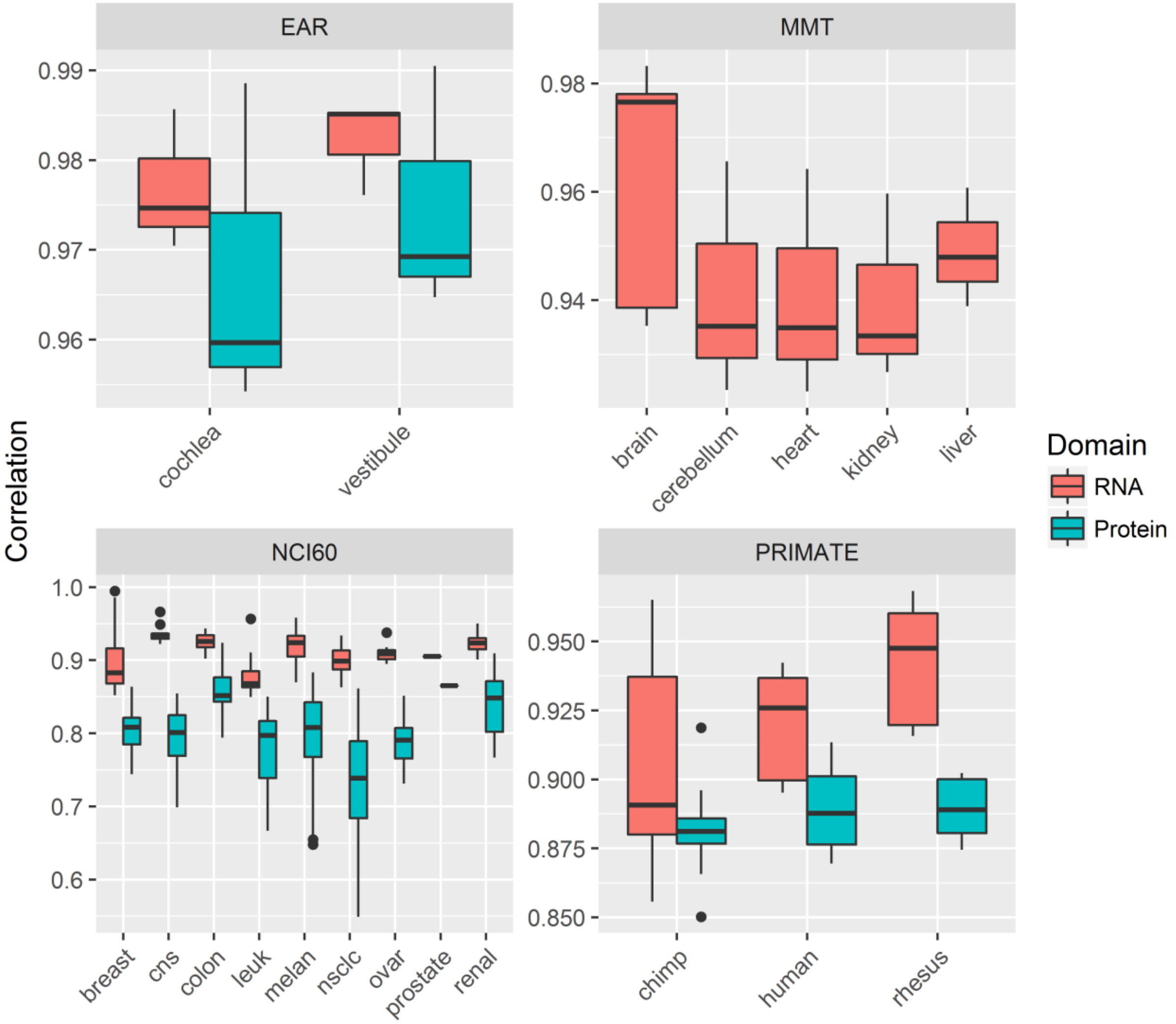


**Figure S4.** Correlation between replicates. By 'replicates' we mean samples from the same group. For each dataset we plotted the boxplots of the distribution of Pearson's correlation (r) between replicates in the mRNA levels (pink) and the protein levels (light blue) aggregated by group. For the MMT dataset we had no protein replicates, so only mRNA correlations are presented. See Fig. 4 for box plot structure. Outliers are not labeled.

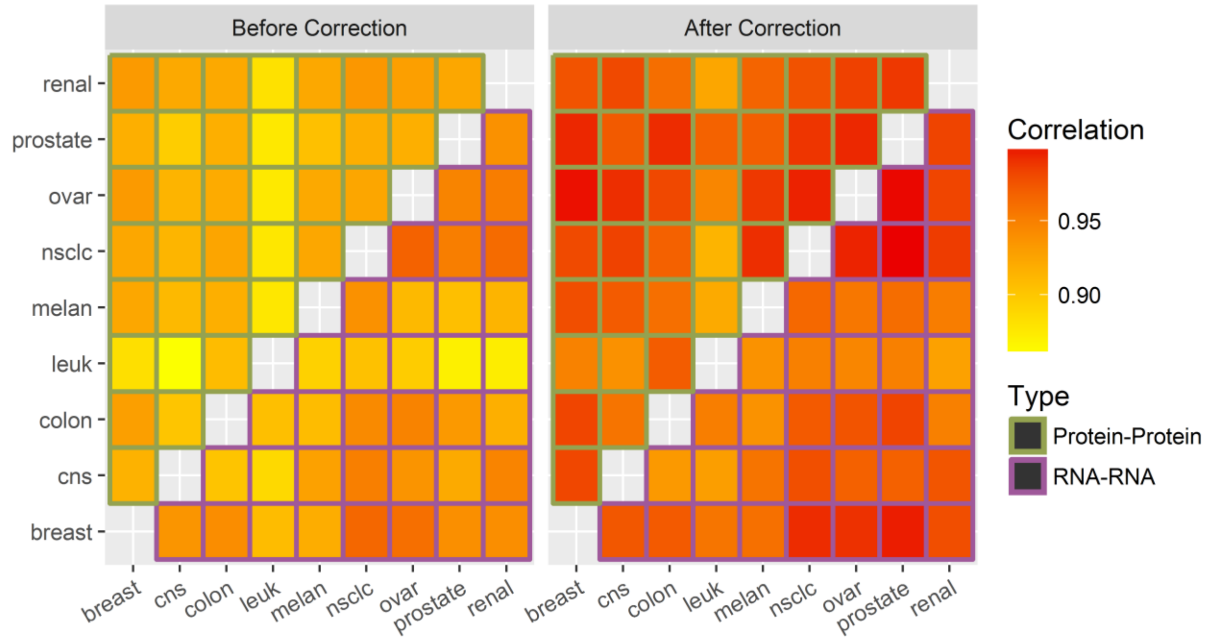


**Figure S5.** Corrected correlation plot for the NCI60 dataset. Left: Pearson's correlation (r) before correction. Right: After applying Spearman’s method to correct for attenuation of protein-protein and mRNA-mRNA correlations. Note that a corrected correlation is not bound by 1. Before applying the correction only 8/36 pairs had higher correlations in the protein domain. After applying the correction the number increased to 24. See caption in Fig. 1 for the structure of the plot.

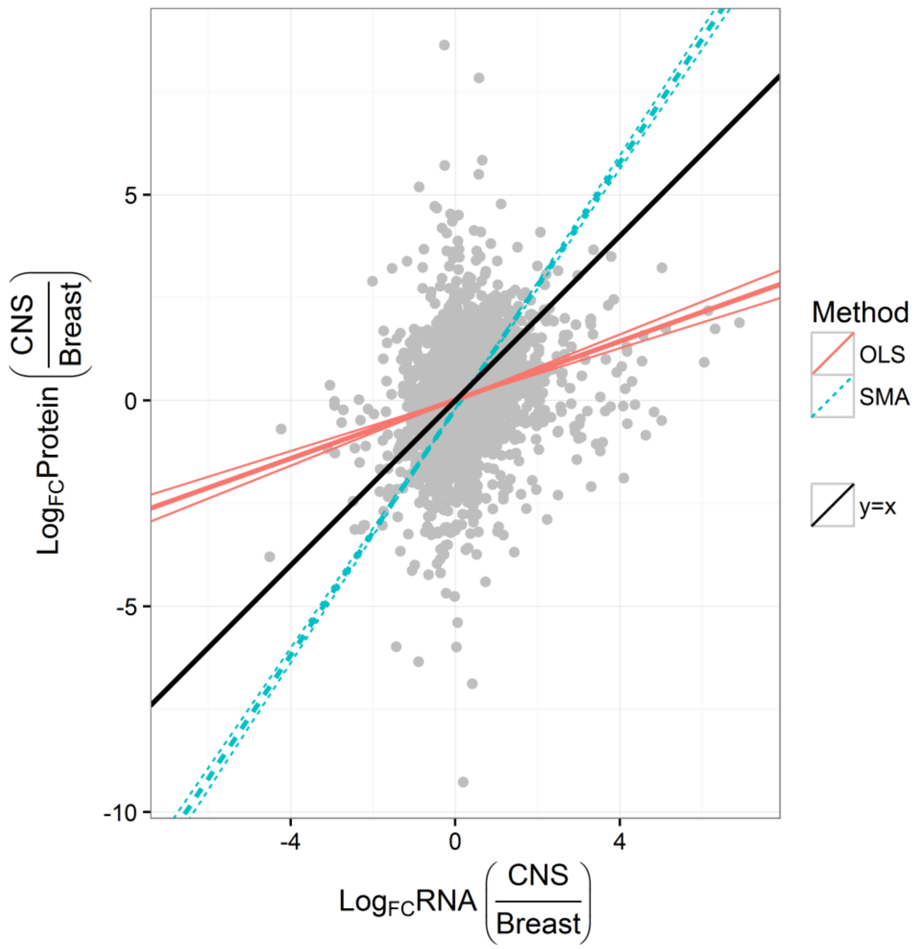


**Figure S6.** Discordance between SMA and OLS regression methods. Comparing the CNS and the breast NCI60 cell lines, the protein fold changes (y-axis) were regressed on the mRNA fold changes (x-axis). The fitted regression lines using either OLS (red, solid) or SMA (blue, dashed) were plotted, along with their 95 percent confidence interval (thinner lines). The black line is y=x. While the OLS slope is significantly lower than 1, suggesting range compression, the more reliable SMA slope is significantly higher than 1, in accordance with range expansion.

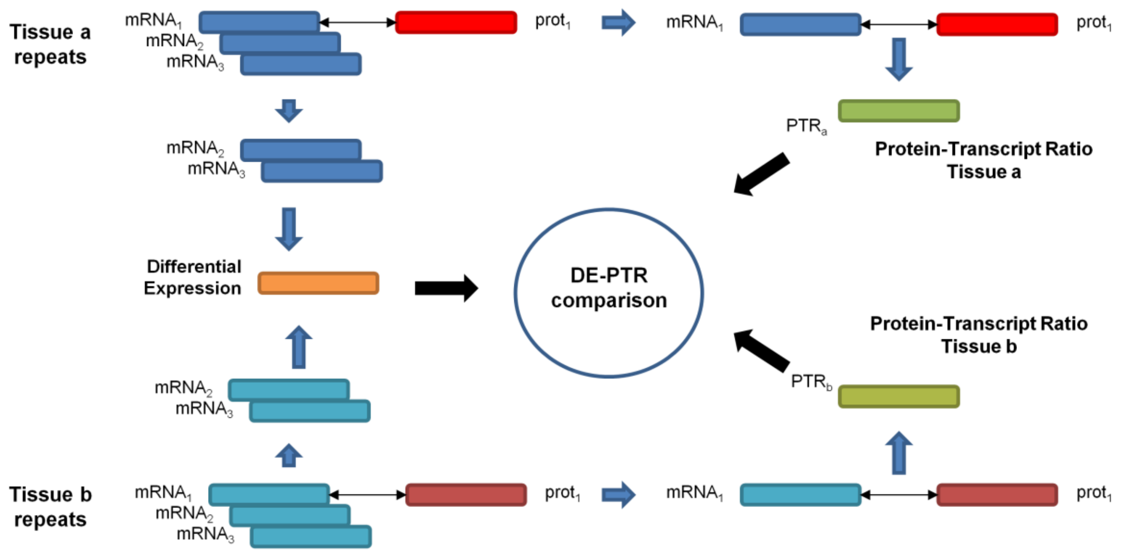


**Figure S7.** Illustration of how mRNA samples are split in order to decouple differential expression analysis from protein-transcript ratios calculation. The latter requires integration with protein data, that can be a matched sample when such pairing exists, or averaged protein levels otherwise. This decoupling is needed for the Wilcoxon test to be unbiased.

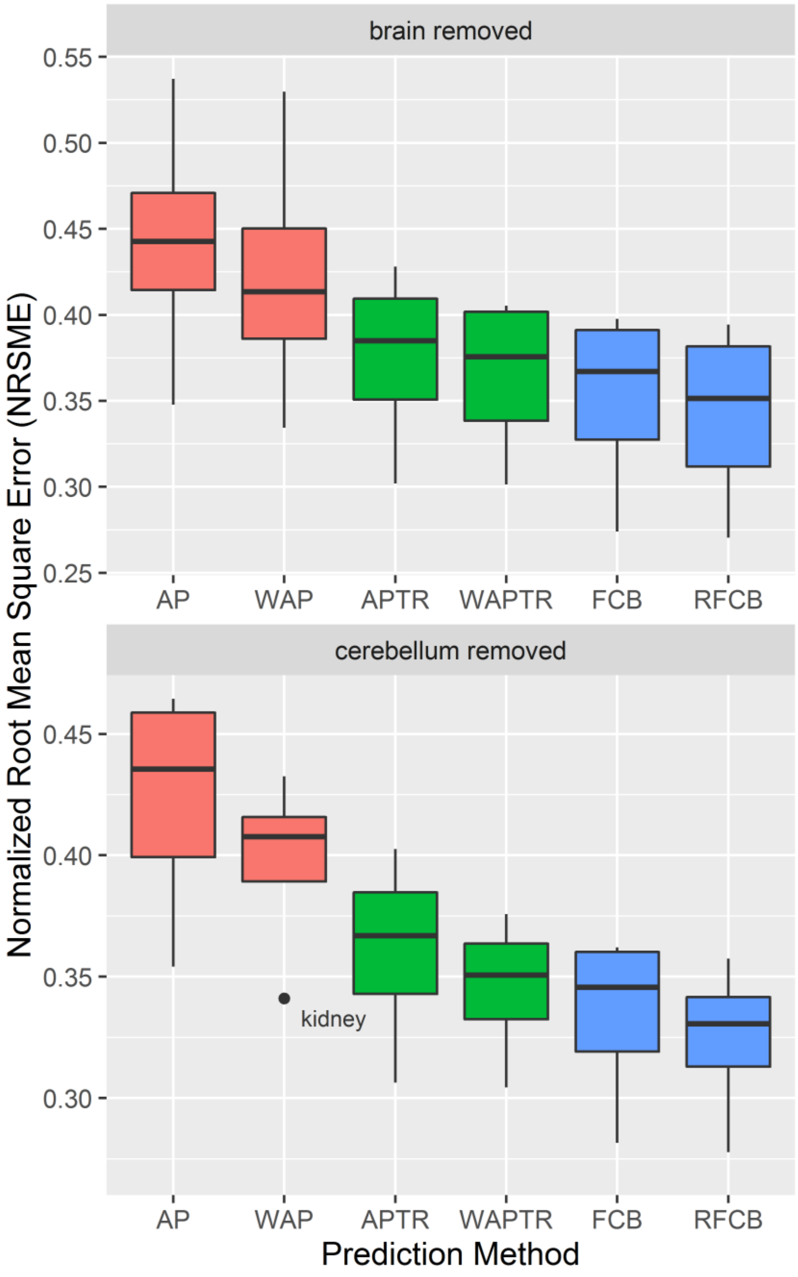


**Figure S8.** Quality of protein level prediction methods on the MMT dataset. Results are shown after the removal of either the brain tissue (up), or the cerebellum tissue (down). For more details see the captions in Fig. 4.

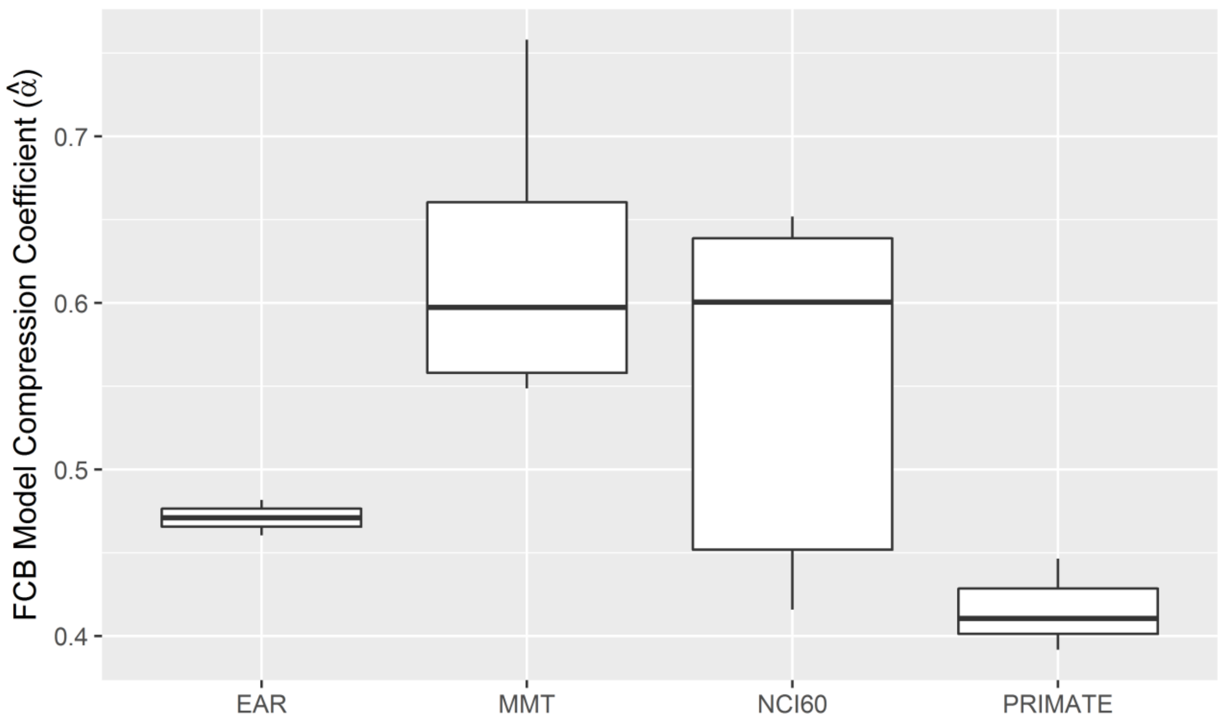


**Figure S9.** Estimated FCB model compression coefficient α. The FCB model was fitted separately for each group using linear regression. The boxplots show the distribution of α in the different datasets. See Fig. 4 for box plot structure.

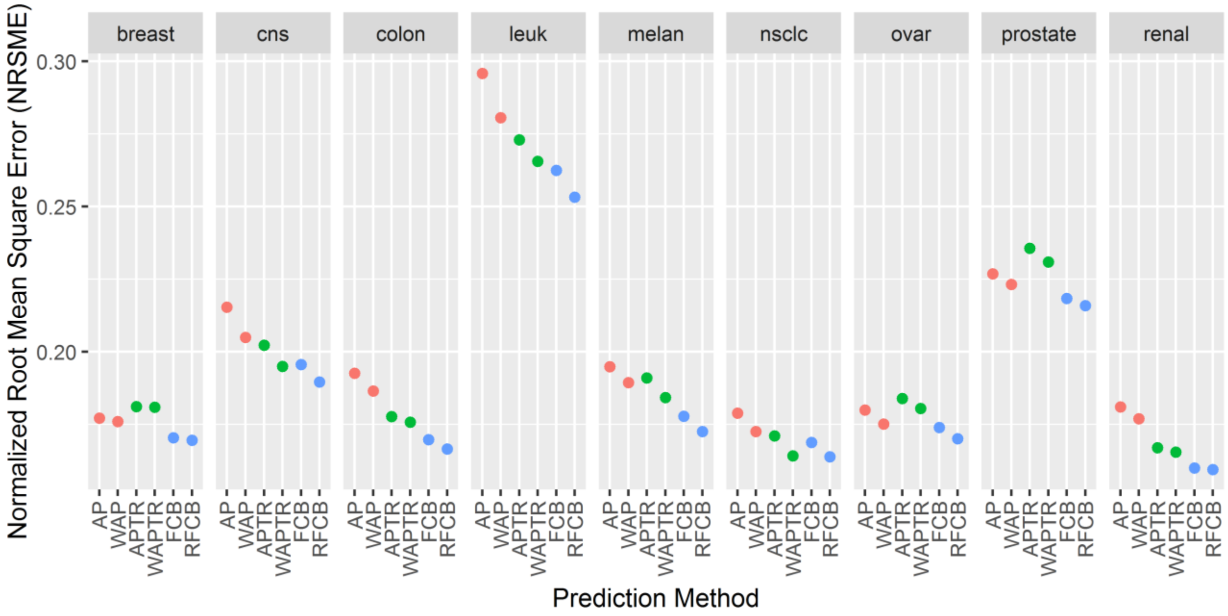


**Figure S10.** Quality of protein level prediction methods in NCI60 groups. For each group, we plotted the normalized root mean square error (NRMSE) in the prediction of protein levels, using six described methods: Averaged Protein (AP), Weighted Average Protein (WAP), Average PTR (APTR), Weighted Average PTR (WAPTR), FC Based (FCB), and Relaxed FCB (RFCB). Each box contains the prediction quality for a single group.

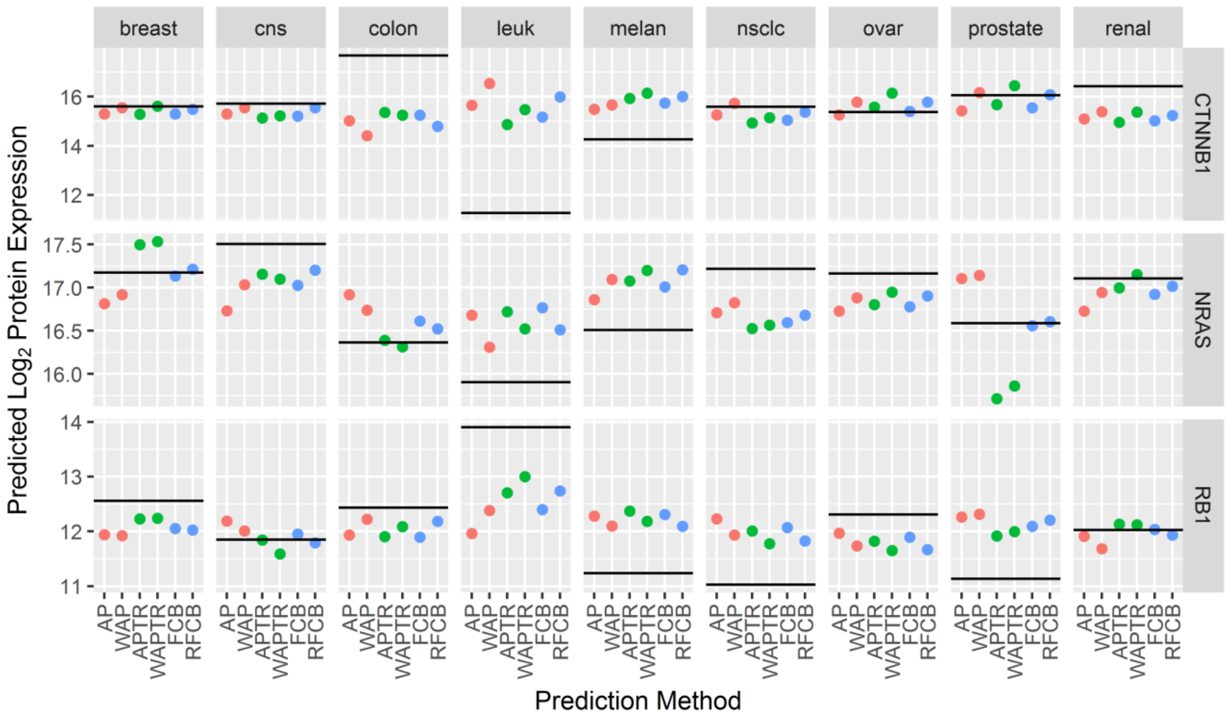


**Figure S11.** Quality of protein level prediction methods for oncogenes in the NCI60 dataset. For each group (columns) and gene (rows), we plotted the prediction of protein levels for that gene, using six described methods (see legend for Figure S10). The true (measured) protein levels are marked by horizontal lines.

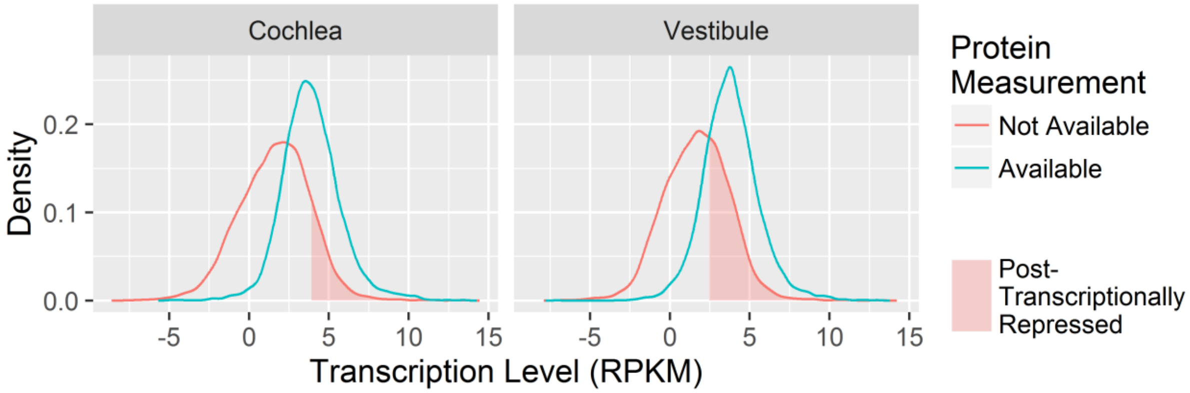


**Figure S12.** Distribution of mRNA levels in the cochlea (right) and vestibule (left). Density plots are shown for the mRNA levels of genes for which measured protein is available (blue), or not available (red). The red area marks the fraction of the distribution where genes with no protein measurement are considered post-transcriptionally repressed (Supplementary Results).

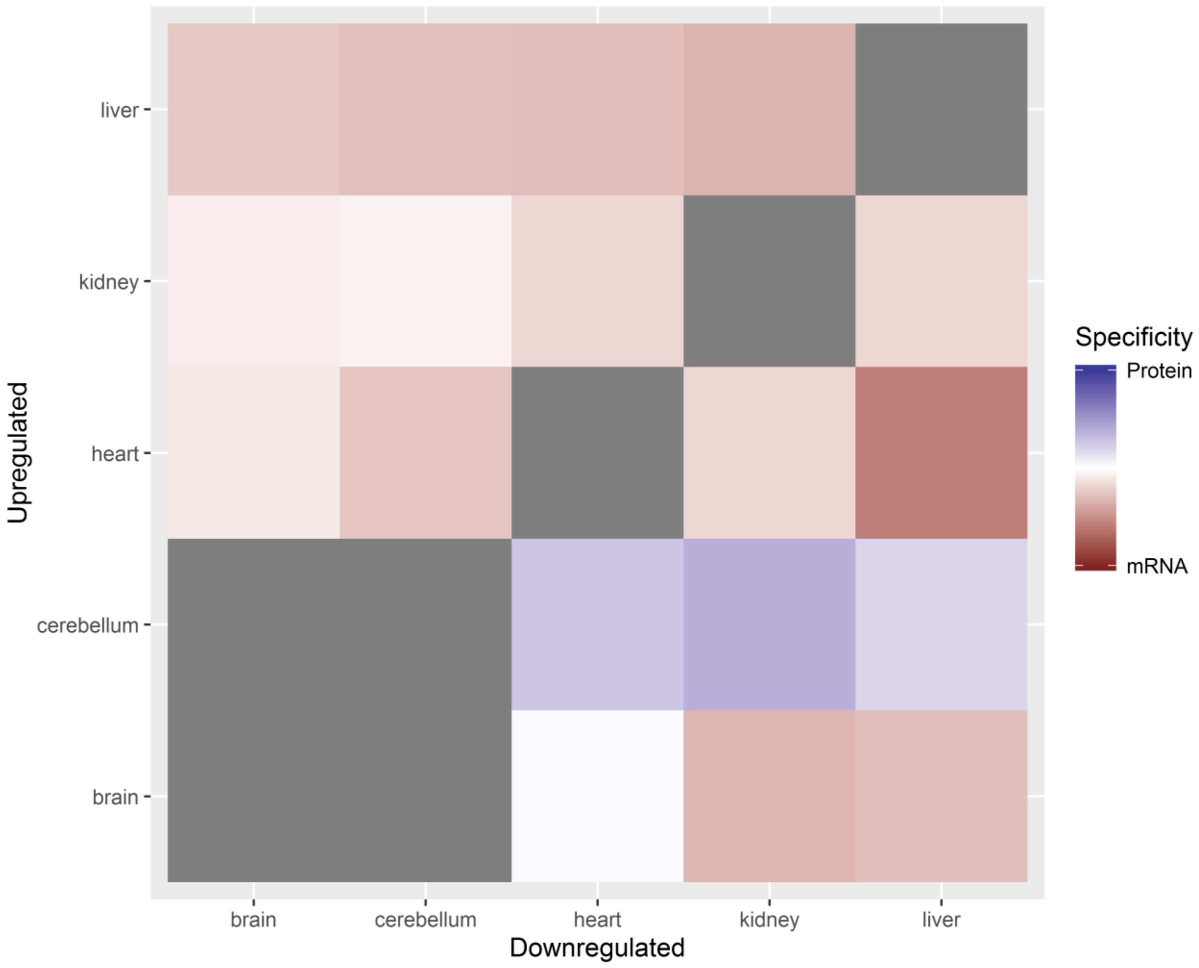


**Figure S13.** Semantic specificity of enrichments to protein or mRNA in the comparison of pairs of tissues [MMT]. Each tile represents a comparison between a pair of tissues in a certain direction, i.e., the terms emerged from genes that are up-regulated in the tissue of the y-axis compared to the tissue of the x-axis. The color of the tile indicates how much the enrichment terms tend to be specific to a single domain, ranging from full specificity to protein (blue) to full specificity to mRNA (red). Pairs, for which no terms were found, are colored in gray. The tiles on the diagonal do not represent a valid comparison.

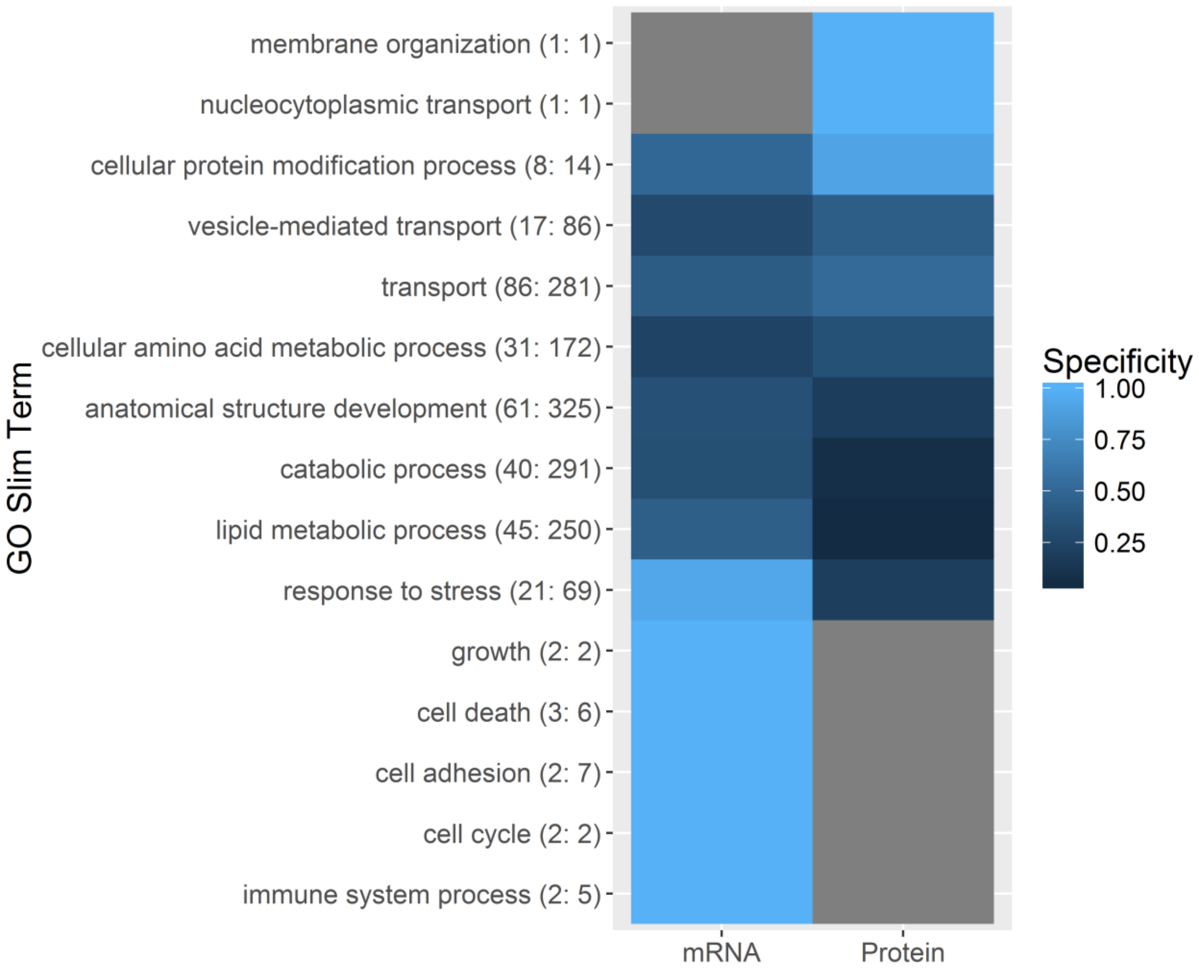


**Figure S14.** Transcriptome versus translatome specificity degrees associated with GO slim terms [MMT]. The heatmap illustrates the specificity of GO slim terms (rows) to the transcriptome and the translatome (columns), where a specificity of 1 indicates that all appearances of the GO term and its descendants are unique to the respective domain, and a specificity of 0 indicates that all these appearances are present in the other domain as well. For each term, the number of unique GO terms that were aggregated and the overall count of their appearances are listed in parenthesis. Included in this figure are only the GO terms for which the proportion of RNA unique terms, out of all unique terms, is significantly different than a background probability of 0.56 (two-sided proportion test, *q-value*≤0.1), or that were spotted only in one of the domains (in such a case the tile of the other domain is colored in gray).

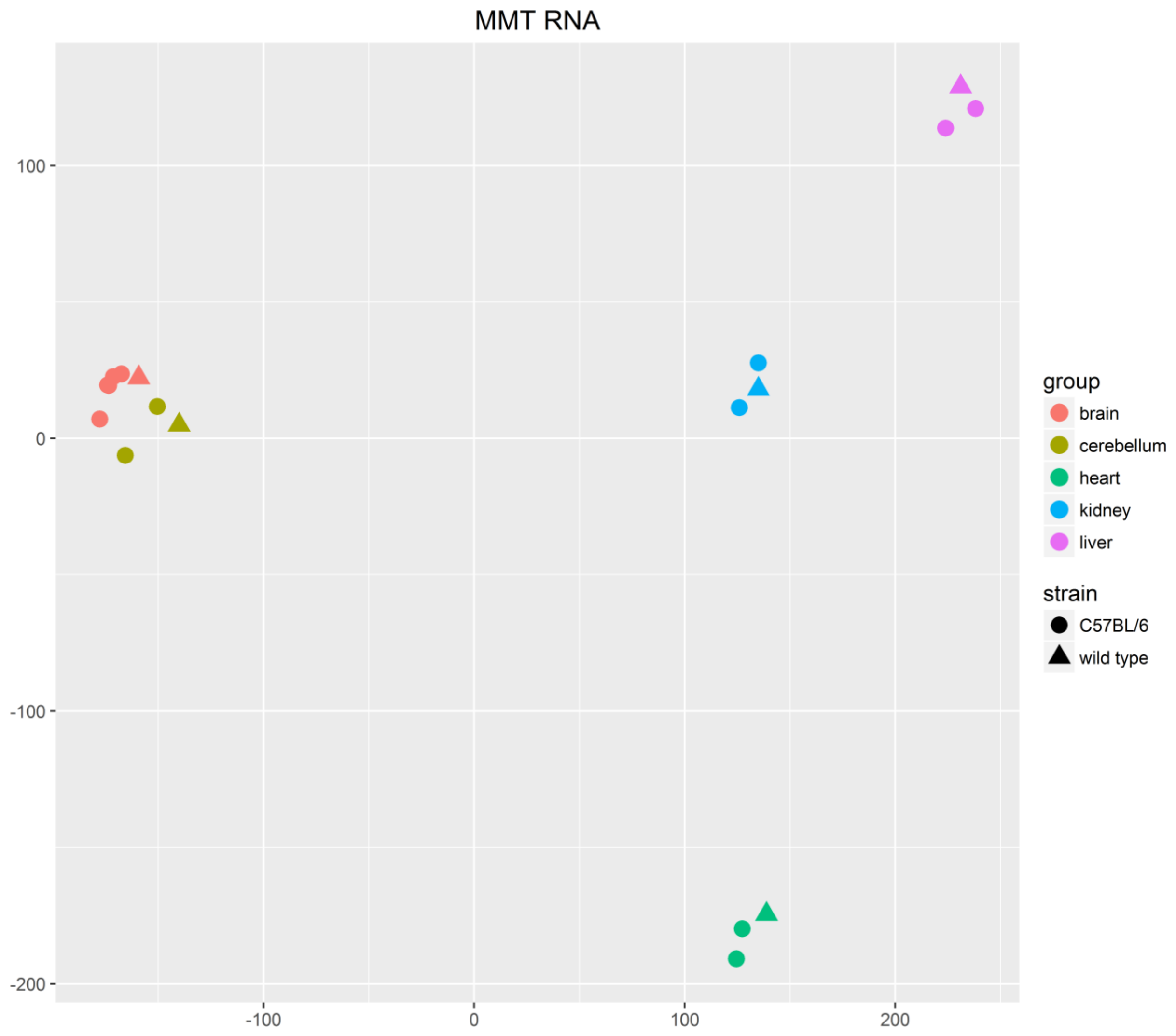


**Figure S15.** MDS plot comparing samples in the MMT dataset according to their mRNA expression. Color indicates the tissue, whereas the shape fits the mouse strain. It is clear that the tissue identity separates the samples better than the strain.

## SUPPLEMENTARY METHODS

### Avoiding biases in correlation measurements

mRNA and protein levels were $\log_2$ -transformed, and averaged across all samples from the same group, disregarding missing values. Correlation was measured between pairs of groups, for mRNA and protein separately. Protein-mRNA correlations for each group were also calculated.

For each pair, correlation was measured between all genes that were expressed in at least one sample of protein, and one sample of mRNA, in both groups. Applying this filter is critical, as lowly expressed genes and proteins suffer from low detection rates, with a higher detection threshold in the protein domain. By applying this filter, we reduced the bias that is caused by the difference in the detection abilities.

Another bias that should be accounted for is the different levels of noise in the mRNA and protein domains. The mRNA replicates are more correlated with each other than the protein replicates in all relevant datasets (Supplementary Result 'Higher correlation between replicates in the mRNA domain'; Figure S4). The higher noise in the protein domain would cause a larger reduction in the observed correlations compared to the real correlations (i.e. before the induction of noise) for protein. To account for this bias, we used Spearman's method to correct for attenuation of correlation, and obtained better estimators for protein-protein and mRNA-mRNA correlations. The effect of the correction is demonstrated in Figure S4 for the NCI60 dataset.

### Choice of variant of major axis

We followed the recommendations in [1] in order to choose which variant of major axis (MA) to use. For a pair of groups, the variance of error of $\log FC_{mRNA}$, $E_{mRNA}$, is approximately the sum of the variances of error of the log-transformed mRNA expression levels in both groups. We make the simplistic assumption that the errors are independently and normally distributed with the same variance across genes and replicates. Hence, within a group, the variance of error in a single measurement can be estimated from replicates, by taking the mean of gene expression levels' variances. To account for the averaging we do over the replicates when calculating gene expression, we divide the variance by the number of replicates in order to get the variance of error in a group. The same can be done to estimate the variance of error of $\log FC_{protein}$, $E_{protein}$. For protein, the estimator $\widehat{E_{protein}}$ would be an underestimate, due to the large number of missing measurements. The variance of the variable $\log FC_{mRNA}$, $V_{mRNA}$, can be estimated by reducing $E_{mRNA}$ from the observed variance. The same can be done to estimate the variance of $\log FC_{protein}$, $V_{protein}$. If the ratio $\widehat{E_{mRNA}}/\widehat{E_{protein}}$ is closer to 1 than to the ratio $\widehat{V_{mRNA}}/\widehat{V_{protein}}$ , MA is preferred over Scaled MA (SMA) and vice-versa. Performing these calculations on our data, we observed that for the EAR and PRIMATE MA is preferred, and for NCI60 SMA is preferred (for most pairs). For

MMT, since there are no replicates in protein, the error cannot be estimated. Assuming that the error in protein for MMT is similar to the error in other MS platforms, the data supports the overall use of MA.

**Non-parametric approach**

We used a nonparametric approach to test whether genes that are up-regulated in one group versus the other in the mRNA domain will show lower PTR in that same group versus the other. This idea allows us to use established tools for differential expression (DE) analysis of mRNA that have high power in discovering such genes.

For each dataset, for each pair of groups, we conducted the following analysis (compare Figure S7):

1. We separated one mRNA sample from each group for PTR calculation.
2. For each group, we took the matching protein sample for PTR calculation. For unpaired datasets (EAR and MMT) we took the average over all protein samples in the group for PTR calculation.
3. For each group we divided the protein levels in the mRNA levels, to get a PTR vector. We will refer to the difference $\log PTR_{group2} - \log PTR_{group1}$ as $\log FC_{PTR}$.
4. Using the remaining mRNA samples, we performed DE analysis. edgeR [2] was used for the analysis in EAR and MMT RNA-seq datasets and samr (https://cran.r-project.org/web/packages/samr, see [3]) was used for the NCI60 microarray-based data, as well as the PRIMATE RNA-seq dataset (as the counts matrix was not directly available). Both methods also output estimations of $\log FC_{mRNA} = \log mRNA_{group2} - \log mRNA_{group1}$ for every gene, referred to as the *DE value*. These estimations differ slightly from those calculated by us in the regression analysis. We chose to work with them, in order for the PTR calculation to be consistent with the DE analysis.
5. We tested the significance of the Spearman's rank correlation between the $\log FC_{PTR}$ and the $\log FC_{mRNA}$ vectors, using the function cor.test in stats package (www.R-project.org). If our buffering assumption is correct, we expect significant negative correlations. We refer to this test as a *global* test.
6. We tried two FDR thresholds (0.05, 0.1) for defining which genes are DE. For each FDR threshold, we tested whether the genes up-regulated in the first group have higher $\log FC_{PTR}$ than the genes in the other group. We used a one-tailed Wilcoxon rank sum test. We refer to this test as a *local* test.

In the DE testing, we excluded genes for which one or both protein values were missing and thus the *PTR* could not be calculated. We repeated this analysis by for every possible pair of repeats selected in step 1 for the PTR calculations. In order to summarize the results we took a median over the results of all

pairs. We report both original p-values and FDR corrected q-values. The correction was applied for each dataset and for each test (correlation, Wilcoxon rank sum with FDR 0.05, and with FDR 0.1) separately, over all pairs of groups.

**EAR enrichment analysis**

For mRNA DE we used the edgeR package, with a detection threshold of $q{-}value \leq 0.05$. For protein we used the samr package, two class unpaired test, with threshold $q{-}value \leq 0.1$. We used a less strict FDR threshold for the protein in order to obtain a large enough list of genes for this analysis. The default parameters of samr allow some missing data imputation using the k-nearest neighbor algorithm. Still, for some genes samr could not assign a test score, and they were removed from the background set. We performed the enrichment analysis using the Expander software [4], checking for enrichment in Gene Ontology (GO) (http://www.geneontology.org) 'biological process' (BP) ontology (*corrected* $p{-}value \leq 0.05$) (see 'Choice of Enrichment Analysis Tool' as to why Expander was used). For mRNA and protein, we looked for enrichments in the set of genes up-regulated in the cochlea versus the vestibule and vice-versa, using as a background set all the genes that passed the filter and were tested for DE.

For comparing lists of enrichments terms we used the REVIGO tool [5]. A threshold of 0.7 was used to define similar terms.

**EAR GOProfile analysis**

The package goProfiles [6] was used to examine whether DE genes are different between the domains. We used the function 'compareGOProfiles' with the default parameters to check if the functional profiles of the genes differ. We began the comparison from a 6th level of the BP ontology, performing a global test for difference of function profiles. If it was significant, we stopped and performed a class-by-class test to identify significant classes (Fisher's exact test with FDR correction, using the function 'fisherGOProfiles', FDR threshold 0.05). If no classes were found we restarted the process at one level higher.

**MMT enrichment analysis**

For each pair of tissues, and each direction of comparison, we calculated $FC_{protein}$ and $FC_{mRNA}$ (for $FC_{protein}$ the relative protein levels were used). We filtered out genes for which we could not calculate either quantity. We created two different orderings of the genes, one by $FC_{protein}$ and the other by $FC_{mRNA}$. We ran GOrilla [7] on both lists, using the 'biological process' (BP) ontology, with an FDR threshold of $1*10^{-3}$ (see 'Choice of Enrichment Analysis Tool' as to why GOrilla was used). This analysis provided us with two lists of terms for each combination of tissues and comparison direction.

In order to determine the semantic similarity between an mRNA list and a protein list of terms, we adopted the method of [8]. The semantic transcriptome specificity is defined as 1 minus the averaged maximal similarities between each term in the mRNA list with any term in the protein list; the semantic translatome specificity is defined as 1 minus the averaged maximal similarities between each term in the protein list with any term in the mRNA list. The semantic similarity between two GO terms is a score between 0 (no similarity) to 1 (full similarity), and it is calculated using the Rel method [9] provided by GOSemSim [10]. The difference between the semantic translatome and transcriptome specificities is a score between -1 to 1.

To calculate the transcriptome versus translatome specificity degree associated to GO slim terms, we followed [8]. For each GO slim term *t* the transcriptome specificity degree is calculated as the ratio between the number of times a descendant term of *t* was mRNA specific to the number of times a descendant term of *t* appeared, across all combinations of tissue pairs and direction. The translatome specificity is calculated similarly, using the protein specific list of terms. These measures are slightly different from [8], in which the aggregation at the level of GO slim was performed after calculating a term specificity scores. This modification provides a more accurate estimator in a scenario with relatively low number of terms. In order to set a significance threshold on the term specificity to one of the domains, we assumed that the unique enrichments we see are randomly sampled, with each enrichment coming from the mRNA domain in probability *p* and the protein domain in probability *1-p*. We estimated *p*=0.56 as the proportion of unique enrichments that were found in the mRNA domain. Then, we asked for each GO slim term whether the corresponding proportion is different than *p* (two-sided proportion test, *q-value*≤0.1). A proportion larger than *p* would indicate transcriptome specificity, and vice versa. This simplistic null hypothesis, made for the sake of the test, ignores the inherent dependence that exists between the uniqueness of mRNA terms and protein terms. The same significant terms were obtained when using a semantic similarity threshold to determine whether a GO term is unique (*threshold*=0.7, Rel method [9]).

**Choice of enrichment analysis tool**

We used different tools to check for functional enrichment in the EAR and the MMT datasets. For the EAR we used the TANGO algorithm in Expander [4]. This algorithm considers the hierarchical tree-like structure of the gene ontology, using it to provide a good estimation of statistical significance for each term, one that takes multiple testing into account. Also, the reported terms are filtered for redundancy.

For the MMT we decided to forfeit these advantages, in favor of an approach that is cut-off independent. In the MMT dataset there are no replicates (in protein), so a gene DE status cannot be assigned a p-value. If we had chosen to use a cut-off dependent tool like Expander, we would have had to set some arbitrary threshold on the fold-changes in order to define target gene sets. This is different from the analysis in the EAR, where we used a statistical threshold on the corrected p-values, and not on a

threshold based on fold-changes. A statistical threshold allows estimating the amount of noise that enters the enrichment analysis, whereas a threshold on the fold-changes would not allow such estimation and would make the enrichment results questionable. For this reason we chose to work with GOrilla [7], to which we entered the genes in order of decreasing fold-changes.

**Post-transcriptionally repressed genes**

In order to find the post-transcriptionally repressed genes of a group, we ordered the genes in decreasing levels of mRNA expression. We then iterated over the list and calculated the fraction of genes that have a valid measurement in the protein domain, out of those that we already iterated upon. For a given value $q$, the index of the last iteration, before which we saw ten fractions in a row above $q$, used as a threshold. All genes appearing before this index that have no protein measurements, were defined as post-transcriptionally repressed. We note that this definition is more robust than the one used [11], as it avoids inclusion of borderline genes.

We then used the Expander software [4] to perform enrichment analysis on these genes, exploring all ontologies, 'biological process' (BP), 'molecular function' (MF) and 'cellular component' (CC) (corrected p−value ≤0.05). As background we chose all genes with expression above the threshold.

We ran this process for all the groups in datasets EAR, MMT, and NCI60. For PRIMATE, we ran the process on the entire dataset without separation into groups, as an internal test showed that the post-transcriptionally repressed functions in LCLs are similar between species, and such separation would only reduce our power in detecting these functions.

We note that between datasets and between groups within the same dataset, there is high variability in the number of genes with mRNA expression above the threshold (Additional file 9: Table S8, Metadata sheet), suggesting that q could be set per group.

**EAR ID conversion and annotation mapping**

Calculation of molecular weight was done from protein sequences retrieved from UniProt.ws (www.bioconductor.org/packages/release/bioc/html/UniProt.ws.html) for canonical sequences and from Uniprot's [12] file 'uniprot_sprot_varsplic.fasta' for isoforms (Additional file 2: Table S1, Metadata sheet). Conversion of protein IDs to Ensembl IDs was done using UniProt.ws. Ambiguities in the conversion were solved by prioritizing an assignment of IDs that will increase the overlap between the mRNA and the protein domains (Additional file 2: Table S1, Metadata sheet). The conversion from Ensembl IDs to Entrez IDs needed for enrichment and GOProfile analyses was done using Bioconductor [13] annotation packages org.Mm.eg.db and org.Hs.eg.db.

**Summary Statistics**

Summary statistics were calculated at the levels of a dataset, a group within a dataset, and a sample, for samples quantified for both RNA and protein (PRIMATE and NCI60 datasets). All statistics were based on genes with some measurements in both protein and RNA. For statistics at the level of a dataset, we first averaged the gene expression by group, and then averaged the expression in different groups. For statistics at the level of a group, we averaged the expression of a gene over the samples in each group. The statistics calculated where the average, range, and standard deviation of the RNA and protein expression values, as well as the correlation between RNA and protein.

**Plotting**

All heat maps, scatter plots, box plots, and histograms were plotted using the ggplot2 package (cran.r-project.org/web/packages/ggplot2). Source data for figures will be provided upon request.

**Alternative polyadenylation**

Using cuffdiff [14] v2.2.1 we calculated the expression of transcript isoforms in units of FPKM (Fragments Per Kilobase per Million mapped reads) from our EAR RNA-Seq data, against the same reference genome as used in mapping. We found 116,684 isoforms mapping to 37,746 genes. We filtered out 32,675 genes for which we did not have a protein measurement in both tissues. Another 108 genes were filtered out because we did not have an mRNA measurement for them in both tissues, in the processed EAR mRNA dataset, i.e., the dataset used throughout this article. We were left with 24,162 isoforms mapping to 4963 genes. Then, for both tissues, we calculated the proportion of each transcript isoform for each gene. Proportions in the vestibule were subtracted from those in the cochlea to determine the differences in isoform usage. The vector of the differences is marked $prop_{c-v}$. It contains values ranging from -1 for isoforms only present in the vestibule, to 1 for isoforms only present in the cochlea. For each isoform, we also determined the presence of miRNA binding sites using package microRNA [15]. For each miRNA separately, we aggregated the sum of $prop_{c-v}$ by gene, over all isoforms containing a binding site for it. The resulting vector for miRNA i will be marked $miRNA_i$. A positive entry in this vector indicates that the matching gene is more susceptible to miRNA regulation in the cochlea than in the vestibule, and vice-versa. The vector entries are also in $[-1,1]$, with 0 marking no difference in susceptibility. As in [16], we treated the presence of a binding site in an isoform as a binary property, ignoring site multiplicity. Finally, for each miRNA i, we regressed $\log FC_{protein}$ on $\log FC_{mRNA}$, treating $miRNA_i$ as a covariate. The fold change data was the same as the one used for the regression analysis. We performed a generalized likelihood ratio (GLR) test, comparing the model with the covariate, to the model without it. We built a model for each of the 553 miRNAs included in the microRNA [15] package, and applied FDR correction to the results. We note that in [16] multiple comparisons were treated using a different technique of bootstrap analysis.

## SUPPLEMENTARY RESULTS

### EAR RNA-seq consistency and accuracy

We compared the results from our previous micoarray-based study [17] to those of the current experiment. We followed the preprocessing stages of the original article: computing expression levels using the robust multiarray average (RMA) method (with a quantile normalization scheme), and filtering probe sets with no ’Present’ calls according to the Affymetrix MAS5 method. The filtering reduced the number of probe sets from 45,101 to 29,539; 27,347 of which were mapped to Ensembl gene IDs using biomaRt [18]. Probe intensities were averaged by tissue. We resolved multiple matching of IDs by taking the median. Finally, we remained with 16,669 genes, out of which 12,994 mapped to genes detected in the RNA-seq experiment, 5048 mapped to *proteins* detected in the cochlea, and 6453 to *proteins* detected in the vestibule in this study.

The mRNA expression levels from [17] correlate well with those of the new one (0.71 for both tissues). They also show good correlation with the protein expression (0.49 and 0.47 for the cochlea and vestibule). However the correlation is not as high as the correlations observed between the RNA-seq data and protein (0.59 and 0.56 for the vestibule and cochlea). The higher correlations observed for the RNA-seq may be due to its higher accuracy.

In the previous study 488 genes were found to be up-regulated in the cochlear system and 1365 in the vestibule system (t.test, $q\text{−}value \leq 0.1$, $FC \geq 1.3$). Using the same FDR and FC thresholds on the new data, we found 1247 and 2004 genes, respectively (edgeR). The studies show high concordance: 295 and 792 genes are up-regulated in the cochlea and vestibule in both studies, respectively. Only 7 were found to be up-regulated in the vestibule in the previous study and in the cochlea in the current study; and only 2 of the opposite directions (ENSMUSG00000005220, ENSMUSG00000058952, ENSMUSG00000033491, ENSMUSG00000079022, ENSMUSG00000028525, ENSMUSG00000026587, ENSMUSG00000050315; ENSMUSG00000074207, ENSMUSG00000021268). The higher number of DE genes in the current study expresses the increased sensitivity of the RNA-seq technology, and the power of the differential expression (DE) tool.

### EAR MS consistency and accuracy

We compared the results from our previous protein mass spectrometry (MS) study [17] to those of the current experiment. In [17], 456 proteins were identified in one or both tissues. In comparison, 7244 proteins were identified in this experiment. We compared how well the two studies agree on the levels over the overlapping proteins. We mapped the protein GI numbers used in [17] to Ensembl gene IDs, using the tables gene2accession and gene2ensembl available on NCBI site (ftp://ftp.ncbi.nlm.nih.gov/gene/DATA, downloaded on December 18, 2015). We resolved multiple matching of IDs randomly. 399 proteins mapped to proteins detected in the MS experiment, 390 mapped

to proteins detected in the cochlea, and 398 to proteins detected in the vestibule in this study. The data from [17] were $\log_2$-transformed.

The protein expression levels from [17] correlates poorly but significantly with this study for the cochlea ($r$=0.15; $p-value$=3.70×10$^{-3}$) but not for the vestibule ($r$=-0.04; $p-value$=0.49). A better correlation is observed when comparing $\log FC_{protein}$ between the studies ($r$=0.23; $p-value$=3.50×10$^{-6}$) for the 388 genes with all four measurements. The increase in the correlation when comparing fold-changes, suggests that the division reduces a study-specific bias factor. For that reason, we used fold-changes in the following comparison with mRNA levels.

Comparing $\log FC_{protein}$ from [17] with $\log FC_{mRNA}$ from this study, the correlation is moderate but significant ($r$=0.24; $p-value$=2.5×10$^{-6}$) for the 389 genes with all four measurements. It is lower than the correlation of protein fold-changes between the studies, as expected from not comparing the same platforms. It is also much lower than the correlation between $\log FC_{protein}$ and $\log FC_{mRNA}$ from this study ($r$=0.59; $p-value$<2.2×10$^{-6}$) for the 380 genes with all six measurements. The higher correlations observed for the proteomics from this study may be due to its higher accuracy.

Another advantage of the current study over [17] is the existence of multiple replicates per tissue. This allows to assess the variation per protein in protein expression between replicates, and to use validated statistical testing, such as t-test, to identify DE genes.

**Comparison of protocols used to collect mRNA and protein data**

Collecting the mRNA and the proteomic data for a dataset from two different published articles, raises the concern that different protocols for sample preparation and source animals will lead to improper results. While this is not the case for the EAR and the PRIMATE datasets, for which the protocols were similar to allow such comparison, the NCI60 and the MMT datasets should be carefully analysed. In the case of NCI60, this concern is alleviated by the genetic identity of the cell lines used in the two experiments, and by the fact that a comparison of proteomic and transcriptomic data was previously made and showed a significant degree of correlation between the two [19]. As for MMT, we included only adult mice samples in the analysis, carefully choosing which samples to include, though not all RNA samples were of the same species as the protein data (C57BL/6J). Also, we attentively handled tissues for which we had to merge several protein samples from different sub-regions of a tissue, to create a sample comparable to the RNA data. Finally, we provide summary statistics for all four datasets to allow this analysis (Additional file 3: Table S2).

**Higher correlation between replicates in the mRNA domain**

We plotted the correlation between the replicates in the protein domain and in the mRNA domain (Figure S3). For all relevant datasets we can see that the mRNA replicates are more correlated with each other than the protein replicates. This was also confirmed by ANOVA testing in the two large datasets of NCI60

and PRIMATE. We treated the correlations between the replicates as the dependent variable, and the group identity and the data type (mRNA or protein) as explaining factors. For both NCI60 and PRIMATE, the estimated data type coefficients are in the direction that support lower correlation between protein replicates (*p−value*=$1.8\times10^{-75}$, $6.4\times10^{-11}$ respectively).

**MMT mRNA correlations are probably higher than protein correlations**

In the main text it was shown that the observed mRNA correlations are higher than their protein counterparts in MMT. It might be that the observed correlations are higher in the protein than in the mRNA, but this is not the case for the real correlations, i.e. before the induction of noise. One such scenario can happen if measurements in the mRNA are noisier than these in the protein domain. We cannot rule this scenario; however, we have strong reasons to believe that the protein levels are noisier, as the measuring methods used in MMT are similar to those used in the EAR and the NCI60 datasets, where this is the case (see previous section). Moreover, the lack of replicates in the protein domain, prevented the noise reduction by averaging, suggesting that the protein levels estimation is even noisier for the MMT dataset than these two other datasets.

The only pair in the MMT dataset for which the mRNA correlation is higher is brain-cerebellum (0.95 versus 0.87). Still, this might be an artifact caused by higher levels of noise in protein measurements. These are also the two highest correlations measured in this dataset, which is not surprising in light of the resemblance in function of these organs.

**Comparing major axis and ordinary least square regression**

For all pairs of groups in all datasets, we regressed $\log FC_{protein}$ on $\log FC_{mRNA}$ using ordinary least square (OLS) or a variant of the major axis (MA) regression (either MA or scaled MA (SMA)). OLS regression is more standard, and the asymmetrical nature of its formulation fits the paradigm of the protein levels being the "output" of the mRNA levels; However, it is more sensitive to regression dilution bias, which can severely lower the estimate of the slope [20]. (S)MA is more robust to noise in any of the axes. Furthermore, the question we are facing concerns the underlying connection of two variables, and thus it is symmetric, and should be evaluated using (S)MA [20]. As for which variant of major axis (MA) to use, we followed the recommendations in [1] (see Supplementary Methods).

Table S3 [Additional file 4] summarizes this analysis. Using OLS all the slopes calculated were significantly less than 1 (*q−value*≤0.01; medians: EAR: 0.47, PRIMATE: 0.27, NCI60: 0.46, MMT: 0.57), consistent with the aforementioned range compression phenomenon. Moving to (S)MA estimates, we find an increase in coefficients across all pairs. For the EAR and PRIMATE datasets all slopes are significantly less than 1 (medians: EAR: 0.90, PRIMATE: 0.40). For the MMT dataset the slopes of all pairs but one are significantly less than 1 (median = 0.71). For the brain-cerebellum pair the slope is 2.43,

significantly higher than 1. For the NCI60 dataset all slopes (median = 1.46) are significantly higher than 1.

Using MA it seems that the range compression is a common phenomenon for pairs of tissues, or species. For cell lines, an opposite phenomenon of range expansion is occurring. We should be cautious with this statement, as the analysis of pairs that are highly similar in their transcriptomic and proteomic profiles is more sensitive to deviations from the assumptions made by the (S)MA analysis. Such highly similar pairs are the cochlea-vestibule in EAR, brain-cerebellum in MMT, and all the pairs in NCI60.

**FCB compression parameter varies between groups**

The value of the compression parameter $\alpha$ of the FCB model is directly linked to the extent of compression ratio between mRNA and protein fold-changes. This parameter was learned separately for each group using linear regression. Its estimated value varied greatly between datasets and between groups within a dataset (Figure S9). Its median value was the highest in the NCI60 (0.6) dataset. A value lower than 1 supports compression between mRNA and protein fold-changes, however, the estimation of $\alpha$ from the data can be severely biased toward a lower value by noise in the mRNA measurements.

**Protein expression prediction power in different datasets**

The error measure presented in Fig. 4 and Figure S8 is normalized to allow the comparison of prediction quality between datasets. According to this measure, all models perform best on the EAR dataset, then on the NCI60, PRIMATE and MMT datasets in decreasing order of performance. In addition, we scored these differences by measuring the extent of variance in protein levels that is explained by the RFCB model in each of the datasets. In the EAR and NCI60 datasets 95.7% and 93.2% were explained respectively, decreasing to 89.8% in the PRIMATE dataset, and only 82.4% in the MMT dataset. The ranking is also the same using the AP model to score the datasets (94.3%, 91.6%, 87% and 65.7% respectively). We can conclude that the task of predicting protein levels, where one is given expression data from a similar tissue (EAR), or under the scenario of cancerous cell lines (NCI60, see next section regarding outliers), is easier than predicting using data from the same tissue but in different species that were separated millions of years ago (PRIMATE, [21]), or from less similar tissues (MMT).

**Cell lines with lower protein expression prediction power**

In the NCI60 dataset, the prediction scores in the prostate and leukemia cell lines were consistently worse across all methods (Fig. 4 and Figure S10). This suggests either a biological deviation of these two types of cancers from the rest, or a stronger experimental noise in the measurements of protein in both. For the prostate, it may also be the low number of samples that renders the estimators inaccurate.

### Missing protein measurements makes it more difficult to compare data between mRNA and protein

A direct comparison of the genes DE on the mRNA and protein levels is not trivial for the following reasons: (1) Levels of noise in each domain is different, affecting the overall accuracy. Data missing at random has a similar effect. (2) There is a detection bias against lowly expressed genes in the protein domain; as a result, proteins with low expression in one or both of the tissues that are being compared are more likely to have more missing measurements across samples. Thus, the power to detect DE proteins with this property is lower. In contrast, the detection bias in the mRNA domain is much smaller, and the power to detect DE transcripts is much less affected by the detection probability.

Resorting to enrichment analysis alleviates the first problem. Failure to see a single gene DE in one domain but not the other, due to noise, is more probable than missing an entire group of genes that share a function and are coexpressed because of noise. The second problem is more complex, as our ability to detect a function in the protein domain depends on the expression levels of the proteins annotated for it; i.e. there is a bias against detecting functions performed mainly by lowly expressed proteins, as these proteins have lower detection probabilities, and are less likely to be classified as DE.

### EAR low abundance proteins have less chance of being observed

Out of the 7018 proteins that have at least one measurement, only 5101 have at least one measurement in both cochlea and vestibule, 4443 have at least two in each tissue, and 3678 were measured in all six samples. The data are clearly not missing at random, as indicated by the increase in median logarithmized protein level across the four groups (19.91, 20.11, 20.28, and 20.63, respectively), consistent with known literature [22]. For the mRNA data this problem is negligible. One of the filtering stages performed in the preprocessing of this data is including only the genes that have one read per million in three or more of the samples. After this stage 14,722 genes remain, out of which 14,693 genes have full data.

Only 201 genes have some measurements in protein, but not in mRNA. For the rest, we can compare the mRNA levels distribution of the genes that have some measurements in protein, to those without a measurement. We did so separately for the cochlea and vestibule (Figure S12), and observed that the levels of genes that have protein measurements is higher than the levels of genes without a measurement ($p\text{−}value<2.2\times10^{-16}$ for both, one-sided Two-Sample Kolmogorov-Smirnov test). The mRNA levels that we are comparing do not correlate perfectly with the protein levels, yet this still supports the 'missing non at random' quality of the protein data.

### Comparing differential expression results using samr

In the main text, we used DE results for the EAR dataset to show that protein profiles are more similar than their RNA counterparts. However, these results were obtained using different DE tools and thresholds for protein and mRNA. Thus, we reran the analysis using the same tool (samr) and the same FDR threshold of 0.1 for both protein and mRNA.

We found 752 and 956 genes up-regulated in the cochlea and vestibule respectively in the mRNA domain, and 46 and 156 genes in the protein domain. 85.1% of the genes found to be differentially expressed (DE) in protein were also DE in the same direction in mRNA, and 3.5% in the opposite direction. The $FC_{mRNA}$ of genes that were up-regulated in one tissue in both mRNA and protein domains, was significantly more extreme than the $FC_{mRNA}$ of the genes that are DE only in the mRNA domain ($q\text{−}value$=$4.8\times10^{-16}$, $5.5\times10^{-40}$, where *group*2 is the cochlea and vestibule respectively, one-sided Wilcoxon rank sum-test; Median FC: 2.72 versus 1.29 in cochlea, 2.29 versus 1.35 in vestibule).

As in the main text, we observed that (i) more DE genes are found in the mRNA domain, (ii) genes found to be DE in protein were usually DE also in mRNA in the same direction, and (iii) genes found to be DE in both domains had more extreme mRNA fold changes than those found to be DE only in mRNA. These findings make less likely the explanation that the power of the DE analysis tool is what causes the differences observed.

**EAR enrichment analysis**

We reran DE using different filters on the number of measurements in the protein domain: (1) No filter (*NF*). (2) At least one measurement (*1M*). (3) At least one, two or three measurements in both tissues (*1MB*, *2MB*, *3MB*; the last one is the complete cases filter).
Comparing the enriched terms after applying the different filters in protein, it appears that the list of terms obtained using the *3MB* filter is nearly inclusive compared to the list of terms obtained using the other filters. It contains almost all the terms appearing in the enrichment list of *NF*, except for ’skeletal muscle tissue development - GO:0007519’,’locomotion - GO:0040011’, 'regulation of cell motility - GO:0010817', and 'regulation of biological quality - GO:0065008'. It also includes all the functions obtained for *1MB* analysis, except for 'regulation of hormone levels - GO:0010817'. And it is almost identical to the one obtained in *2MB*, except for ’regulation of cell motility' again, and also ’cell-substrate junction assembly - GO:0007044’.

Performing the same comparison for the mRNA enrichment, the *NF* analysis provides a very comprehensive list of 37 enriched terms in the cochlea, and 78 in the vestibule. The size of the list decreases when moving to stricter filters (13 and 38, 4 and 22, 5 and 12, and 3 and 9; For the *1M*, *1MB*, *2MB*, and *3MB* filters respectively). 77% and 87% of the terms enriched in cochlea and vestibule respectively in *1M* analysis emerge also in *NF* analysis. The corresponding numbers for *1MB*, *2MB*, and *3MB* are 75% and 73%, 60% and 83%, and 33% (1/3) and 78%. This suggests that many but not all the

functions found are represented in the *NF* analysis. Specifically, the terms ’nucleobase catabolic process - GO:0046113’ and ’taxis - GO:0042330’ that are enriched in the cochlea in the *1M* analysis and the terms ’secondary metabolic process - GO:0019748’, ’response to stimulus - GO:0050896’, ’regulation of plasma lipoprotein particle levels - GO:0097006’, and ’regulation of coagulation - GO:0050818’ that are enriched in the vestibule in the same analysis, do not appear in the *NF* analysis. The vestibular enriched terms ’biological regulation - GO:0065007’, ’retinoic acid metabolic process - GO:0042573’, and terms related to renal system development (GO:0072001, GO:0032835) appear in the *1MB* analysis but neither in *NF* nor in *1M* analyses. We avoided reporting differences when a similar term was found according to REVIGO.

Summarizing data from all filters, for the protein domain, we see that the only enrichment in the cochlea is related to its role in sensory perception (GO:0007600), whereas the vestibule shows enrichment for general terms related to development and morphogenesis (GO:0009653, GO:0000904, GO:0048468, GO:0048731), more organ-specific terms doing with bone morphogenesis (GO:0060351) and skeletal muscle development (GO:0007519), and less expected terms relating to biological adhesion (GO:0022610), response to wounding (GO:0009611), phagocytosis (GO:0006909) and locomotion (GO:0040011). Regulation of hormone levels (GO:0010817), biological quality (GO:0065008), cell motility (GO:2000145), and cell adhesion (GO:0045785) are enriched regulatory processes.

In the mRNA domain, the cochlear enrichment fits the cochlea’s role in sensory perception (GO:0007600). Often enriched categories are connected to tissue morphogenesis (GO:0048729) and cell development (GO:0048468) with an emphasis on neuronal cell development (GO:0045685, GO:0014014, GO:0007417, GO:0048663, GO:0050768, GO:0045664, GO:0045665). Other terms relate to different aspects of development, such as organ development (GO:0007423, GO:003527, GO:0031016), developmental processes (GO:0032502, GO:0051094), and anterior/posterior pattern specification (GO:0009952). Many are related to cell-cell signaling and cell communication (GO:0023052, GO:0007267, GO:0010646, GO:0035637), including the use of neurotransmitters (GO:0006836) and the serine/threonine kinase signaling pathway (GO:0090092). Others are related to behavioral patterns (’taxis - GO:0042330’ and ’behavior GO:0007610’). Nucleobase catabolic process (GO:0046113) also represents a function enriched in the genes up-regulated in the cochlea. Terms related to signaling were completely absent in the *3MB* analysis, perhaps due to relative low expression of the proteins associated with these categories.

The vestibular enrichment terms include response to stimulus or stress (GO:0050896, GO:0080134) as well as specific responses to wounding, organic substance or other organism, and inflammatory responses (GO:0009611, GO:0010033, GO:0051707, GO:0006954). Other terms related to the immune response include ’antigen processing and presentation - GO:0019882’, ’B cell receptor signaling pathway - GO:0050853’, ’leukocyte chemotaxis - GO:0030595’ etc. The list contains general signaling related terms (GO:0007267, GO:0023052), as well as those of more specific signaling pathways: ’regulation of

protein secretion - GO:0050708', 'regulation of hormone levels - GO:0010817', and 'regulation of cAMP metabolic process - GO:0030814'. The positive regulation of peptidyl-tyrosine phosphorylation, a similar metabolic function, is also enriched (GO:0050731). Some terms are related to morphogenesis (GO:2000027, GO:0009653), development (GO:0032502), cell proliferation (GO:0042127), and cell death (GO:0010941). Few terms are related to biological adhesion (GO:0022610, GO:0016337), extracellular matrix organization (GO:0030198), and tissue remodeling (GO:0034103). There are terms related to vasculature development and blood circulation (GO:0001944, GO:0008015). Regulation of plasma lipoprotein particle levels (GO:0097006) is an enriched metabolic process. Locomotion (GO:0040011) is also an enriched term. In this tissue too, terms related to signaling are not present in the *3MB* analysis, as are immune related terms.

**GO profiles comparison in EAR**

Comparing the sets of terms emerging in enrichment analysis is sensitive to the significance threshold set for the analysis. In order to gain another perspective on the difference in functions between the mRNA and the protein, we used goProfiles [6], which enabled us to ask whether the functional profiles of the DE genes are different between the domains without presuming a threshold on term significance. A functional profile is defined here as the joint frequencies of annotation in a given set of GO classes.

For each tissue, we compared the functional profile of the genes up-regulated in the protein domain, with the functional profile of the genes up-regulated in the mRNA domain using goProfiles [6]. We followed the methodology described in [6] by starting the comparison in a deep level of the GO ('biological process', 6th level), performing a global test for difference of function profiles, and only if it was significant, doing a class-by-class test to identify significant classes (see "Material and Methods"). If such were not found we restarted the process at a level less deep.

For the cochlea, the result of the global test was insignificant ($p{-}value>0.05$) for all levels. For the vestibule, the result of the global test was significant in all levels 3-6 ($p{-}value$s=$6.0\times10^{-4}$, $6.0\times10^{-4}$, $5.0\times10^{-4}$, $3.6\times10^{-3}$), however only in level 3 we got a significant class, in the class-by-class test, which was 'cell adhesion - GO:0007155' ($q{-}value=1.75\times10^{-2}$). 16.0% of the genes in the mRNA domain were annotated by this GO term or a descendant term, whereas 26.3% of the genes in the protein domain were annotated for it (30 genes were common, 27 unique to the mRNA domain, and 11 to the protein domain; Additional file 7: Table S6). The percentage of annotated genes in the protein analysis that were not DE in the mRNA domain is 26.8%; a higher percentage than the background probability of 23.7%, but insignificantly ($p{-}value>0.05$, one-sided Fisher exact test). The fact that in deeper levels we got a significant result in the global test but not in the class-by-class test is due to the higher power of the global test.

In conclusion, we could not reject the possibility that the ’functional profile’ of the genes up-regulated in the cochlea is the same for mRNA and protein. For the genes up-regulated in the vestibule, not only did we reject this null hypothesis, but we also managed to pinpoint the difference on the cell adhesion category, for which 26% of the genes in the protein were annotated, and only 16% of the genes in the mRNA domain.

**Some tumor related functionalities are controlled through post-transcriptional repression**

Kwon et al. analyzed genes with high mRNA expression, with not even a single measurement in the protein domain [11]. To conduct a similar test on our datasets, for each group separately, we found the lowest mRNA expression level above which at least a fraction $q$ of the genes have a valid measurement in the protein domain, with some correction for robustness. We defined the post-transcriptionally repressed genes as those above the mRNA threshold but with no protein measurement. We then performed enrichment analysis for each group, comparing the post-transcriptionally repressed genes to the background of all genes above the mRNA threshold.

We wanted to set a single fraction $q$ for all datasets and groups. In Kwon et al. $q$=0.8 was used [11]. To accommodate the sparsity of our data we chose $q$=0.65. Figure S12 shows the EAR thresholds. For the cochlea and vestibule the thresholds were 3.97 and 2.58 RPKM, respectively, and 3603 and 7880 genes (out of 14,722) had higher expression, respectively. In both tissues, about one third of these genes were identified as post-transcriptionally repressed, as dictated by the parameter $q$. For other datasets see Additional file 9: Table S8, Metadata sheet.

We checked the post-transcriptionally repressed genes for enrichments, against the background of all genes exceeding the threshold (Additional file 9: Table S8). Some recurrent terms were intuitively explained as artifacts of a detectability bias (see next section). In the NCI60 data, other terms, such as ’cellular response to interferon-gamma - GO:0071346’ in the colon, were more group specific. It was previously shown that colon cancer cell lines with Ki-ras mutations display reduced expression of interferon (IFN)-responsive genes [23]. It is known that IFN-γ regulates the mRNA translation of components of the immune system [24,25]. Combining the two, we can explain why the cellular response to IFN-γ is post-transcriptionally repressed in colon cancer. The regulation of the immune response is also post-transcriptionally repressed in ovarian cancer according to our analysis. Several pathways are known to cause immune suppression in this cancer type [26–28]. Our findings suggest that some of the suppression is post-transcriptionally mediated. Other interesting terms are ’structural constituent of ribosome - GO:0003735’ and ’organellar ribosome - GO:0000313’ in leukemia [NCI60]. Indeed, in leukemia [29], as well as in ovarian cancer [30], the levels of expression of some ribosomal protein genes were found to be positively correlated with favorable clinical course. This suggests that reduction in certain ribosomal proteins is contributing to the progression of cancer. We conclude that this reduction is

achieved by post-transcriptional repression. The term ’embryonic organ development - GO:0048568’ appears in multiple cancer lines, such as breast, CNS, colon and NSCLC. It was reported that in poorly differentiated cancers, including breast and CNS tumors, the gene expression signature is similar to the one in embryonic stem cells [31]. We conclude that post-transcriptional repression is involved in this process. Many of the enrichments found in the PRIMATE datasets are related to either the mitotic cell cycle or to lymphocyte homeostasis and the immune response. This suggests the involvement of post-transcriptional repression in the immortalization process used to establish LCLs [32]. The EAR and MMT data suggest several other functions that are post-transcriptionally repressed, but additional evidence is required to support these hypotheses (see section 'Post-transcriptional repression in EAR and MMT datasets).

### Protein detection bias leads to false-classification of processes as post- transcriptionally repressed

In the enrichment analysis of post-transcriptionally repressed genes, the term ’intrinsic to membrane - GO:0031224’ is persistent across all groups in the EAR and MMT enrichments. Moreover, the frequencies of the genes annotated with it are very high across all groups (>28.0%). This observation most probably represents a problem in isolating the membrane-bound proteins [33], and not an *in vivo* phenomenon. This term does not arise in the NCI60 analysis, perhaps due to the simplicity of working with cell lines, which allows a better extraction of membrane-bound proteins. The presence of terms related to receptors, especially those membrane-bound, can also be explained this way.

Another term that is consistent, also in the NCI60, is ’DNA binding - GO:0003677’. Similar terms that appear are related to sequence specific binding, zinc ion binding (GO:0008270), and positive and negative regulation of gene expression. The low detectability of the proteins annotated for these classes can be explained by overall lower abundance of transcription factors.

### Post-transcriptional repression in EAR and MMT datasets

We cannot always distinguish between post-transcriptional repression and detection bias. The two terms ’extracellular region - GO:0005576’ and ’humoral immune response - GO:0006959’ enriched in the post-transcriptionally repressed genes in the liver [MMT] might represent a translational repression of the humoral immune system in a healthy tissue, or a technical problem in obtaining extracellular proteins, even in a tissue where such are heavily expressed [34]. The term ’WW domain binding - GO:0050699’ in the kidney [MMT] refers to a modular protein domain that is found in many signaling pathways [35], and is involved in the pathogenesis of renal syndromes such as polycystic kidneys [36] and Liddle’s syndrome [37,38]. It is possible that post-transcriptional repression plays a part in signaling regulation in the kidneys through the WW domain. However, this enrichment does not necessarily represent an *in vivo*

phenomenon, as 3/10 of the genes enriched for this category are components of the epithelial sodium channel [39], and their protein products may suffer from low detectability. Another term for which the distinction is difficult is ’regulation of bone mineralization - GO:0030500’ in the vestibule [EAR]. Bone ossification of the bony labyrinth is essential for proper inner ear development [40], whereas ossification of the membranous labyrinth due to infection can cause hearing loss and vertigo [41]. Translational repression might be needed to prevent ossification of the membranous labyrinth. Still, only a low frequency of genes is enriched for this function (0.76%), rendering this discovery less persuasive.

**Reaffirming conclusions of PRIMATE study**

For the PRIMATE dataset the buffering of transcriptional differences at the protein level was already shown [42]. First, the authors showed that the number of DE genes between species is by far larger in the mRNA domain than the protein, supporting a strong compensatory selection pressure on the protein domain. Then, they developed technique to find genes that have higher interspecies variation in one of the domains, and showed that most of the genes they found using it, had higher interspecies variation in the mRNA domain than in the protein domain. This led them to conclude that the difference in the number of DE genes could not be attributed to a high within-species variation in protein.

In the main text we tried to address some methodological limitations in [42]: (i) The identification of genes with higher within-species variation in one domain was performed on all genes and not just those that are DE. It is still possible that for genes that are DE the within-species variation in protein is generally higher than the within-species variation in mRNA. (ii) This identification was done using an ad-hoc method, with no clear definition of the null and alternative hypotheses, and no estimates for its false positive and negative rates. (iii) The authors’ usage of asymptotic statistical tests clearly does not fit their scenario due to the small number of samples. In this study, by using more appropriate statistics, we reaffirm the correctness of the observation.

**Possible explanations to how coordination of translation and transcription is achieved**

The buffering is achieved by changing the translation rates, the protein degradation rates, or both. We speculate that the translation rate is the factor that is changing between the two tissues (main text). It has been suggested that translational efficiency decreases with increased mRNA levels due to competition for scarce resources, e.g., ribosomes [16]. However, as ribosomes are part of a nonspecific translation machinery, that would not work slower in translating a specific gene if it is over-transcribed. Certain proteins may participate in replication and transcription (e.g., Rap1 and Abs1 in yeast), they could be incorporated into the mRNA, exported from the nucleus, and differentially affect the rate of translation at the ribosome [43], although no proof was provided for this mechanism. Moreover, it was not explained how the coordination of translation and transcription is achieved. Yeast transcription and mRNA

degradation rates were demonstrated to be negatively correlated, and showed that the mutations responsible for this effect usually influence both transcription and mRNA degradation [44]. Analogously, we believe that epigenetic changes are those coordinating the rates of transcription and translation in our system.

**Alternative polyadenylation fails to explain protein fold changes**

It was reported in [16] that alternative polyadenylation (APA) can explain much of the variation in the $\log FC_{protein}$ in the scenario of adipogenic differentiation. Many protein coding genes have multiple polyadenylation sites. APA results in subpopulations of transcripts differing in 3'UTR length. Shorter versions can contain less miRNA binding sites, and be less susceptible to miRNA regulation. The authors fitted a linear model, regressing $\log FC_{protein}$ on $\log FC_{mRNA}$ . By adding the effect of only two-miRNAs to the model, the variance explained by it increased from 15.5% to 32.1%.

We followed the methodology described in [16] in order to find miRNAs that are responsible for significant differences in translation efficiencies between the EAR tissues, mediated by alternative polyadenylation events (see Supplementary Methods). A different isoform usage, between the cochlea and the vestibule, was observed for 19,242 transcripts of 3539 genes. For these transcripts the differences in isoform usage between the two tissues, $prop_{c-v}$, is centered around 0, with a standard deviation of $4.7\times10^{-2}$. Three genes were associated with transcripts with $prop_{c-v} > 0.5$. The gene Serpinb5 is transcribed almost exclusively (99.7%) to ENSMUST00000112729 in the vestibule, and is transcribed almost exclusively (97.22%) to ENSMUST00000086701 in the cochlea. We note that this gene is DE as it expressed 27.7 fold higher in the cochlea (edgeR, q−value≤0.05,). The gene Serpinb6, which belongs to the same protein family, is involved in deafness [45]. The gene Espin-Like (Espnl) is transcribed almost exclusively (96.7%) to ENSMUST00000088904 in the cochlea, whereas in the vestibule 36% is transcribed to ENSMUST00000088904 and 64% to ENSMUST00000176156. The homologue Espin was found to be involved in epithelial morphogenesis, stereociliogenesis and postnatal maturation of the inner ear [46]. The main product of the gene Ank1 in the vestibule is ENSMUST00000141784 (57.5%), but only a negligible fraction of this transcript is found in the cochlea. This gene is related to mechanotransduction in the inner ear [47]. Espin and Ank1 gene are especially interesting, as their different splice variants have varying 3'UTR lengths, and thus might harbor different miRNA binding sites.

For each miRNA *i* and for every gene, we calculated the difference in the proportion of target isoforms between the two tissues. We treated the resulting vector, $miRNA_i$, as a covariate in the regression of $\log FC_{protein}$ on $\log FC_{mRNA}$. No miRNA significantly improved over the base model (GLR test, q−value≤0.1). The miRNA associated with the lowest p−value is mmu-miR-409-3p. Incorporating the effect of this miRNA, resulted in an increase of 0.13% in the variance explained, compared to the base

model. In [16] the incorporation of the best explaining miRNA, resulted in an increase two orders of magnitude higher. A single miRNA targeted on average 171.4±33.6 different genes, of which 123.8±25.2 show different isoform usage. We conclude that the incorporation of a miRNA with this average number of targets into the model, can explain, at most, the unexplained variance of 2.5% of the observations. This might call for the use of a different method that will allow the incorporation of multiple miRNAs in the model, without significantly increasing the number of tests being performed.

In conclusion, we tried to use the same method as in [16] in our EAR data, and failed to see a substantial effect. It seems that APA is much less common in this dataset, so this result is not very surprising. It was previously shown that the miRNAs work primarily by degrading the transcript and not by translational repression [48]. Our lack of findings fits this hypothesis. Yet, the small sample size and our naive assumptions on the miRNA regulation are also to blame.

**REFERENCES**

1. Legendere P. Model II regression user's guide, R edition. R Vignette. 1998;

2. Robinson MD, McCarthy DJ, Smyth GK. edgeR: a Bioconductor package for differential expression analysis of digital gene expression data. Bioinformatics. 2010;26:139–40.

3. Tusher VG, Tibshirani R, Chu G. Significance analysis of microarrays applied to the ionizing radiation response. Proc. Natl. Acad. Sci. U. S. A. 2001;98:5116–21.

4. Ulitsky I, Maron-Katz A, Shavit S, Sagir D, Linhart C, Elkon R, et al. Expander: from expression microarrays to networks and functions. Nat. Protoc. 2010;5:303–22.

5. Supek F, Bošnjak M, Škunca N, Šmuc T. REVIGO summarizes and visualizes long lists of gene ontology terms. PLoS One. 2011;6:e21800.

6. Salicrú M, Ocaña J, Sánchez-Pla A. Comparison of lists of genes based on functional profiles. BMC Boinformatics. 2011;12:401.

7. Eden E, Navon R, Steinfeld I, Lipson D, Yakhini Z. GOrilla: a tool for discovery and visualization of enriched GO terms in ranked gene lists. BMC Bioinformatics. 2009;10:48.

8. Tebaldi T, Re A, Viero G, Pegoretti I, Passerini A, Blanzieri E, et al. Widespread uncoupling between transcriptome and translatome variations after a stimulus in mammalian cells. BMC Genomics. 2012;13:220.

9. Schlicker A, Domingues FS, Rahnenführer J, Lengauer T. A new measure for functional similarity of gene products based on Gene Ontology. BMC Bioinformatics. 2006;7:302.

10. Yu G, Li F, Qin Y, Bo X, Wu Y, Wang S. GOSemSim: an R package for measuring semantic similarity among GO terms and gene products. Bioinformatics. 2010;26:976–8.

11. Kwon T, Huse HK, Vogel C, Whiteley M, Marcotte EM. Protein-to-mRNA ratios are conserved between Pseudomonas aeruginosa strains. J. Proteome Res. 2014;13:2370–80.

12. Magrane M, Consortium U. UniProt Knowledgebase: a hub of integrated protein data. Database (Oxford). 2011;2011:bar009.

13. Gentleman RC, Carey VJ, Bates DM, Bolstad B, Dettling M, Dudoit S, et al. Bioconductor: open software development for computational biology and bioinformatics. Genome Biol. 2004;5:R80.

14. Trapnell C, Hendrickson DG, Sauvageau M, Goff L, Rinn JL, Pachter L. Differential analysis of gene regulation at transcript resolution with RNA-seq. Nat. Biotechnol. 2013;31:46–53.

15. Gentleman R, Falcon S. microRNA: Data and functions for dealing with microRNAs. 2012.

16. Spangenberg L, Correa A, Dallagiovanna B, Naya H. Role of alternative polyadenylation during adipogenic differentiation: an in silico approach. PLoS One. 2013;8:e75578.

17. Ulitsky I, Shamir R. Identifying functional modules using expression profiles and confidence-scored protein interactions. Bioinformatics. 2009;25:1158–64.

18. Durinck S, Spellman PT, Birney E, Huber W. Mapping identifiers for the integration of genomic datasets with the R/Bioconductor package biomaRt. Nat. Protoc. 2009;4:1184–91.

19. Moghaddas Gholami A, Hahne H, Wu Z, Auer FJ, Meng C, Wilhelm M, et al. Global proteome analysis of the NCI-60 cell line panel. Cell Rep. 2013;4:609–20.
20. Smith RJ. Use and misuse of the reduced major axis for line-fitting. Am. J. Phys. Anthropol. 2009;140:476–86.
21. Goodman M, Porter CA, Czelusniak J, Page SL, Schneider H, Shoshani J, et al. Toward a phylogenetic classification of Primates based on DNA evidence complemented by fossil evidence. Mol. Phylogenet. Evol. 1998;9:585–98.
22. Csárdi G, Franks A, Choi DS, Airoldi EM, Drummond DA. Accounting for experimental noise reveals that mRNA levels, amplified by post-transcriptional processes, largely determine steady-state protein levels in yeast. Snyder M, editor. PLOS Genet. 2015;11:e1005206.
23. Klampfer L, Huang J, Corner G, Mariadason J, Arango D, Sasazuki T, et al. Oncogenic Ki-ras inhibits the expression of interferon-responsive genes through inhibition of STAT1 and STAT2 expression. J. Biol. Chem. 2003;278:46278–87.
24. Su X, Yu Y, Zhong Y, Giannopoulou EG, Hu X, Liu H, et al. Interferon-γ regulates cellular metabolism and mRNA translation to potentiate macrophage activation. Nat. Immunol. 2015;
25. Goñalons E, Barrachina M, García-Sanz JA, Celada A. Translational control of MHC class II I-A molecules by IFN-gamma. J. Immunol. 1998;161:1837–43.
26. Barnett B, Kryczek I, Cheng P, Zou W, Curiel TJ. Regulatory T cells in ovarian cancer: biology and therapeutic potential. Am. J. Reprod. Immunol. 2005;54:369–77.
27. Yang R, Cai Z, Zhang Y, Yutzy WH, Roby KF, Roden RBS. CD80 in immune suppression by mouse ovarian carcinoma-associated Gr-1+CD11b+ myeloid cells. Cancer Res. 2006;66:6807–15.
28. Patankar MS, Jing Y, Morrison JC, Belisle JA, Lattanzio FA, Deng Y, et al. Potent suppression of natural killer cell response mediated by the ovarian tumor marker CA125. Gynecol. Oncol. 2005;99:704–13.
29. Dürig J, Nückel H, Hüttmann A, Kruse E, Hölter T, Halfmeyer K, et al. Expression of ribosomal and translation-associated genes is correlated with a favorable clinical course in chronic lymphocytic leukemia. Blood. 2003;101:2748–55.
30. Dua K, Williams TM, Beretta L. Translational control of the proteome: relevance to cancer. Proteomics. 2001;1:1191–9.
31. Ben-Porath I, Thomson MW, Carey VJ, Ge R, Bell GW, Regev A, et al. An embryonic stem cell-like gene expression signature in poorly differentiated aggressive human tumors. Nat. Genet. 2008;40:499–507.
32. Hussain T, Mulherkar R. Lymphoblastoid Cell lines: a Continuous in Vitro Source of Cells to Study Carcinogen Sensitivity and DNA Repair. Int. J. Mol. Cell. Med. 2012;1:75–87.
33. Barrera NP, Robinson C V. Advances in the mass spectrometry of membrane proteins: from individual proteins to intact complexes. Annu. Rev. Biochem. 2011;80:247–71.

34. Gruys E, Toussaint MJM, Niewold TA, Koopmans SJ. Acute phase reaction and acute phase proteins. J. Zhejiang Univ. Sci. B. 2005;6:1045–56.
35. Bork P, Sudol M. The WW domain: a signalling site in dystrophin? Trends Biochem. Sci. 1994;19:531–3.
36. Varelas X, Miller BW, Sopko R, Song S, Gregorieff A, Fellouse FA, et al. The Hippo pathway regulates Wnt/beta-catenin signaling. Dev. Cell. 2010;18:579–91.
37. Schild L, Lu Y, Gautschi I, Schneeberger E, Lifton RP, Rossier BC. Identification of a PY motif in the epithelial Na channel subunits as a target sequence for mutations causing channel activation found in Liddle syndrome. EMBO J. 1996;15:2381–7.
38. Staub O, Gautschi I, Ishikawa T, Breitschopf K, Ciechanover A, Schild L, et al. Regulation of stability and function of the epithelial Na+ channel (ENaC) by ubiquitination. EMBO J. 1997;16:6325–36.
39. Loffing J, Schild L. Functional domains of the epithelial sodium channel. J. Am. Soc. Nephrol. 2005;16:3175–81.
40. Rinkwitz S, Bober E, Baker R. Development of the Vertebrate Inner Ear. Ann. N. Y. Acad. Sci. 2006;942:1–14.
41. Cureoglu S, Schachern PA, Rinaldo A, Tsuprun V, Ferlito A, Paparella MM. Round window membrane and labyrinthine pathological changes: an overview. Acta Oto-Laryngologicaryngologica. 2005;125:9–15.
42. Khan Z, Ford MJ, Cusanovich DA, Mitrano A, Pritchard JK, Gilad Y. Primate transcript and protein expression levels evolve under compensatory selection pressures. Science. 2013;342:1100–4.
43. Tuller T, Kupiec M, Ruppin E. Determinants of protein abundance and translation efficiency in S. cerevisiae. PLOS Comput. Biol. 2007;3:e248.
44. Dori-Bachash M, Shema E, Tirosh I. Coupled evolution of transcription and mRNA degradation. PLOS Biol. 2011;9:e1001106.
45. Sirmaci A, Erbek S, Price J, Huang M, Duman D, Cengiz FB, et al. A truncating mutation in SERPINB6 is associated with autosomal-recessive nonsyndromic sensorineural hearing loss. Am. J. Hum. Genet. 2010;86:797–804.
46. Sekerková G, Zheng L, Mugnaini E, Bartles JR. Differential expression of espin isoforms during epithelial morphogenesis, stereociliogenesis and postnatal maturation in the developing inner ear. Dev. Biol. 2006;291:83–95.
47. Corey DP, Sotomayor M. Hearing: Tightrope act. Nature. 2004;428:901–3.
48. Guo H, Ingolia NT, Weissman JS, Bartel DP. Mammalian microRNAs predominantly act to decrease target mRNA levels. Nature. 2010;466:835–40.
